# Nutrient Sensing via Gut in *Drosophila melanogaster*

**DOI:** 10.3390/ijms23052694

**Published:** 2022-02-28

**Authors:** Gouri Chopra, Shivam Kaushik, Pinky Kain

**Affiliations:** Regional Centre for Biotechnology, NCR Biotech Science Cluster, Faridabad 121001, Haryana, India; gouri.chopra@rcb.res.in (G.C.); shivam.kaushik@rcb.res.in (S.K.)

**Keywords:** enterocytes, *Drosophila*, gut-brain axis, gustatory receptors, enteroendocrine cells

## Abstract

Nutrient-sensing mechanisms in animals’ sense available nutrients to generate a physiological regulatory response involving absorption, digestion, and regulation of food intake and to maintain glucose and energy homeostasis. During nutrient sensing via the gastrointestinal tract, nutrients interact with receptors on the enteroendocrine cells in the gut, which in return respond by secreting various hormones. Sensing of nutrients by the gut plays a critical role in transmitting food-related signals to the brain and other tissues informing the composition of ingested food to digestive processes. These signals modulate feeding behaviors, food intake, metabolism, insulin secretion, and energy balance. The increasing significance of fly genetics with the availability of a vast toolbox for studying physiological function, expression of chemosensory receptors, and monitoring the gene expression in specific cells of the intestine makes the fly gut the most useful tissue for studying the nutrient-sensing mechanisms. In this review, we emphasize on the role of *Drosophila* gut in nutrient-sensing to maintain metabolic homeostasis and gut-brain cross talk using endocrine and neuronal signaling pathways stimulated by internal state or the consumption of various dietary nutrients. Overall, this review will be useful in understanding the post-ingestive nutrient-sensing mechanisms having a physiological and pathological impact on health and diseases.

## 1. Introduction

**Figure 1:**
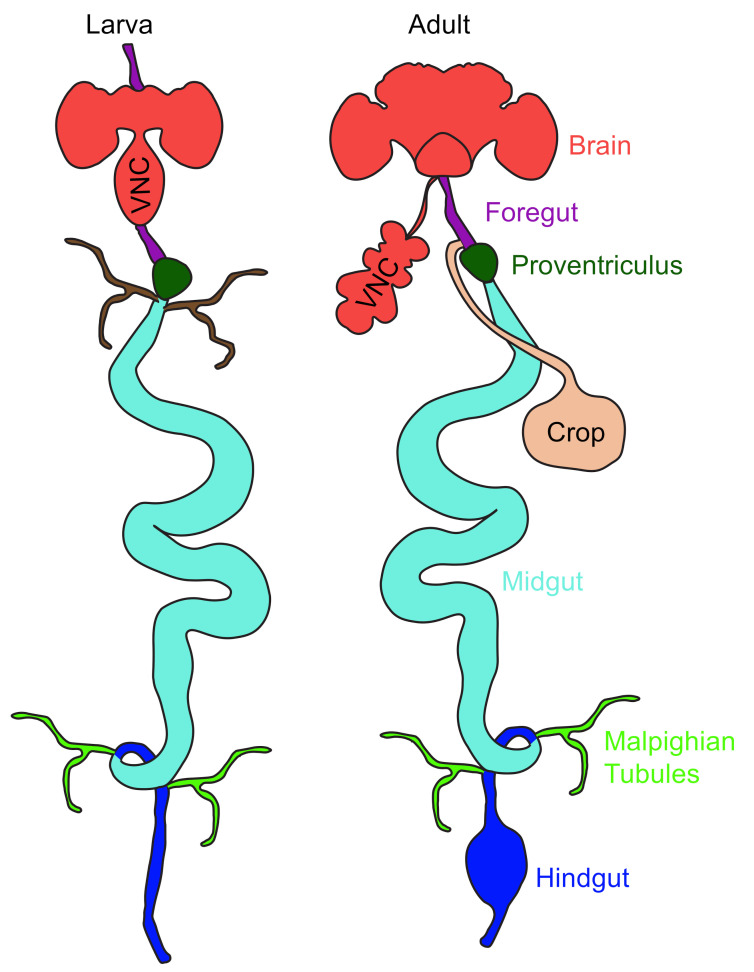
The fly gut during the larval and adult stages is divided into the foregut, the midgut, and the hindgut. The foregut comprises of pharynx, esophagus, and proventriculus. The crop (in adults only) stores the food ingested by the flies. The food is then pushed into the midgut, which is the main site of digestion. The midgut opens into the hindgut, where the residue of the midgut is mixed with the extract of malpighian tubules are blind-ended ducts that mainly play a role in osmoregulation and excretion.

Nutrients are simple organic molecules that, after digestion, are engaged in biochemical reactions that produce energy in animals. The part of the nervous system located in an animal’s gut involved in digestion and absorption of nutrients is known as the enteric nervous system (ENS). It is a lesser-explored system compared to the central nervous system (CNS) in human health and diseases. The animal’s gut or the “second brain” communicates with the brain through secreted chemicals and neural circuits. The growing importance of gastrointestinal (GI) signals in the regulation of food intake, insulin production, and peripheral nutrient storage has sparked an interest in studying how the gut senses and responds to nutritional information. Invertebrate model system *Drosophila melanogaster* shares a homologous gut system with that of mammals and provides an ideal situation to study nutrient sensing via gut (Figure 1). The foregut in the fly (esophagus in humans) passes the consumed food to the crop (a food reservoir organ; called stomach in humans), where storage and digestion of food occurs. Absorption of nutrients takes place in the anterior midgut (small intestine in humans). Further absorption of water and electrolytes takes place in the hindgut (large intestine in humans). Lastly, excretion takes place via anus [1].

*Drosophila* gut is lined by an epithelial monolayer consisting of four cell types: intestinal stem cells (ISCs), absorptive enterocytes (ECs), secretory enteroendocrine (EE) cells, and enteroblasts (EBs), which, along with gut chemosensors, allow the nutrient uptake in the organism. Apart from nutrient absorption, the GI tract detect nutrients and activate events involving the whole endocrine system and neural components that talk to the brain and other tissues to maintain metabolic homeostasis. In the post-ingestion sensing system, the GI tract plays an important role in the interaction between the host and a meal, informing the brain about the nutrient composition, food texture, and meal size. Altogether, nutrient-sensing mechanisms through the gut as well as the effects of the postprandial rise in glucose, lipids, and amino acids, are vital for energy and glucose homeostasis via direct and indirect neural and endocrine mechanisms in various organs. Though the glucose-sensing pathways in other tissues also impact metabolic regulation, but we will not be reviewing them here [2,3,4].

Recently, the GI tract has been recognized as a major source of signals modulating feeding behaviors, food intake, metabolism, insulin secretion, and energy balance. Growing evidence suggests that the gut-brain axis (GBA) plays a vital role in maintaining mental health and affects feeding behavior when nutrient detection or absorption by the gut is altered. The enteric microbiota also impacts the GBA and causes abnormal nutrient sensing by interacting locally with the intestinal cells, ENS, and CNS through neuroendocrine and metabolic pathways. The bidirectional communication between the brain and digestive tract are thus interesting avenues to explore post-ingestive mechanisms, especially nutrient sensing via gut in health and disease. In this review, we are discussing the importance of the gut in nutrient sensing (digestive/absorptive functions), its neural connectivity with the brain, as well as its role in regulating food preferences by using inter-organ signaling, and its role in various diseases. The compiled understanding of nutrient sensing by the gut could lead to the discovery of physiologically significant intestinal nutrition sensors and the development of new therapeutic targets for diabetes, obesity, aging, neurodegenerative diseases, and GI illnesses.

### 1.1. Structure of the Fly Gut

The *Drosophila* gut contains a simple epithelium that is surrounded by muscles, nerves, and trachea. Further, the GI tract of the flies is divided into foregut, midgut, and hindgut (Figure 1) [5,6,7]. The epithelium of the foregut and the hindgut is lined by a permeable cuticle on the apical side, while the epithelium of the midgut is protected by a peritrophic matrix. The cuticle is shed and renewed after each molt. In adult flies, the midgut is further divided into six anatomical regions (R_0_ to R_5_). Each of these regions can be identified as they are separated by a narrow epithelial boundary [8,9] and have well-defined metabolic and digestive functions.

The food ingested by the fly enters the pharynx, a part of the foregut (Figure 1). The foregut comprises of pharynx, esophagus, and proventriculus. It is then transported via the esophagus by peristaltic movement and stored in an extensible part of the gut called the crop. The food is then pushed into the midgut or the ventriculus by the pumping action of the proventriculus. The cells of the midgut are involved in the secretion of digestive enzymes in addition to neural peptides. The midgut opens into the hindgut, where the residue of the midgut is mixed with the extract of malpighian tubules. Malpighian tubules are blind-ended ducts that mainly play a role in osmoregulation and excretion (Figure 1) [10].

Flies have different GI tracts in each stage of life that supersede each other. The embryonic gut gives rise to the larval gut, which is superseded by a transient pupal gut followed by an adult gut [11,12,13]. The larval and pupal gut form meconium and expel from the body after eclosion. The structural organization of the adult and larval GI tract is predicted to be distinct due to different dietary habits [14], especially the existence of the crop in adults alone (Figure 1). In contrast to the larvae, which feed constantly on solids in order to grow, the adults prefer liquid and feed less frequently.

Most of the nutrient absorption takes place in the midgut of the flies, while ionic and water homeostasis is maintained in the hindgut. The fly’s intestine is made up of an epithelial monolayer that contains ECs and EEs. The ECs are polyploid cells that are involved in nutrient absorption and secretion of digestive enzymes. The secretion of digestive enzymes differ throughout the GI tract. This suggests that the food that enters the midgut is acted upon by different enzymes in different anatomical regions of the midgut and is processed sequentially. The EE cells that interspersed among the ECs act as chemical sensors that produce regulatory peptides upon detection of luminal nutrients [15,16]. Another type of cells present along the basement membrane are ISCs that divide symmetrically and asymmetrically to replace old cells with new ones [17,18]. The activity of the stem cells is influenced by the metabolic state of the organism and also by various environmental factors [12,19,20]. In the fed state, the activity of ISCs increase, which drives tissue growth [21]. The activity of ISCs may also increase in the presence of a toxic agent or a pathogenic microorganism to ensure regeneration of the compromised gut [20,22,23]. This thus indicates that the activity of the intestinal progenitor cells is largely dependent on the nutrient uptake by the organism.

### 1.2. The Gut–Organ Communication

The gut is also responsible for maintaining homeostasis within the fly by communicating with other organs via neuronal and endocrine signals.

#### 1.2.1. Neuronal Communication

The GI tract receives innervations from three different sources and relay signal to three distinct regions of the gut. The stomatogastric nervous system, corpus cardiacum, and neurons of the CNS extend their axon toward the digestive tract, sending signals to the gut for coordinating various gut functions [24,25,26,27,28,29,30,31]. The stomatogastric nervous system relays information to the esophagus, crop, and anterior midgut in adults and to the esophagus in larvae. The innervations in the gut are restricted to three regions: the anterior portion of the gut that comprises the pharynx, esophagus, crop, and the anterior midgut; the midgut-hindgut junction, and the posterior hindgut [27,30,31,32,33]. Most of the enteric neurons during the larval stage of the fly are considered to be motor neurons responsible for coordinating the activity of visceral muscles. The posterior region of the hindgut is innervated by sensory neurons. In adult flies, a similar pattern is observed, and the majority of the innervations are believed to be efferent that control the peristaltic movement while the sensory innervations also exist [30]. The neurons that innervate the gut are chemically diverse and produce different neuropeptides, which have distinct functions modulating neuronal activity. The hindgut in the larvae contains neurites positive for pigment dispersing factor (PDF), ion transport peptide (ITP), and proctolin [29,34,35,36,37]. The midgut in the adults contain neurites positive for serotonin, adipokinetic hormone (Akh), myosupressin, allatostatin (AST), diuretic hormone-44 (Dh44), FMRFamide, short neuropeptide F (sNPF) [28,31,38,39,40]. Six peripheral neurons are found on the proventriculus that express gustatory receptor Gr43a, which functions as a fructose receptor in the CNS [41,42,43]. These neurons extend their dendrites into the lumen of the foregut while their axons innervate the midgut and esophagus towards subesophageal ganglion suggesting possible roles in gut-brain nutrient signaling.

The enteric neurons play a major role in regulating peristalsis and facilitating intestinal transit. This can be inferred by the presence of muscle valves in all the three gut regions innervated by the neurons. The neural activity is responsible for the generation of peristaltic waves and their frequency. However, myogenic activity causes the propagation of the peristaltic waves. This can be inferred as many regions of the GI tract lack neuronal innervations [30]. Recently, it has been suggested that the peristalsis and the availability of nutrients have a direct link. This has been proved by the presence of six neurosecretory cells in the pars intercerebralis of the adult brain that are responsible for secreting Dh44 in response to nutritive sugars [40]. Tachykinin (Tk), another EE cell-derived peptide, regulate peristalsis in the midgut region of the GI tract [44]. These studies indicate that in both adult and larval midgut, enteric innervations are chemically diverse in nature [31,38,39,40,45,46], having important roles in nutrient sensing.

The intestinal monolayer allows only selective compounds, nutrients, electrolytes, and water to pass through it. This selective intestinal permeability is controlled by enteric neurons. Inactivation of these neurons result in abnormal structure of the proventriculus, increased permeability of the gut, which increases the risk for various bacterial infections [47]. The enteric neurons also affect the intestinal trachea and its branching [48]. The branching of the trachea is observed to be dependent on nutritional availability. Such response to nutrients during the larval stages of the life cycle plays a vital role in how the flies respond to malnutrition during the later stages of life.

Recently epithelial role of gut-innervating neurons in the control of fluid balance, has been revealed [30,49]. Silencing of CNS HGN1 (hindgut neuron1) neurons that innervate hindgut and rectum results in an increased defecation rate. They are also shown to be required for the post-mating changes in intestinal fluid retention due to their epithelial innervations. Apart from the role of interneurons (IN1) [50], whose activity is exquisitely dependent on the amount and duration of feeding post pharynx in the GI tract, the contribution of sensory innervation to nutritional homeostasis remains to be investigated.

The enteric neurons also control the amount of food that can be ingested by the fly. In adults, nutrient scarcity causes a hyperphagic response in transgenic flies upon inactivation of insulin-producing neurons. Silencing of insulin-like peptide (Ilp7) neurons innervating midgut increase feeding responses [30,51]. Knockdown of Ilp2 neurosecretory cells that innervate the foregut and synapse with Ilp7 neurons cause reduced food intake. The pharyngeal sensory neurons send information to interneurons whose activity depends on feeding duration and amount [50]. Another function of the enteric neurons is regulation of defecation behavior [52], osmoregulation by malpighian tubule [33], and differentiation of intestinal cells. Down-regulation of secreted signaling proteins such as hedgehog (Hh) reduces the ISC and EC differentiation [53]. Recently, the role of *Drosophila* Piezo in volume-based control of meal size has been discovered. Fly neurons expressing Piezo innervate the anterior gut and crop and respond to tissue distension in a Piezo-dependent manner. Activating Piezo neurons reduce appetite, while *Piezo* mutants and Piezo neuronal silencing causes gut bloating and increases both food consumption and body weight [54].

Post-ingestive sensory feedback from the gut has been assumed to inhibit feeding based on studies performed in blowflies. Severing the recurrent nerve or the medial abdominal nerve, which transmits information from the gut to the brain, results in overconsumption in blowflies [55]. In flies severing the recurrent nerve elevates consumption of sucrose but not water or bitter-tasting solutions [56]. Although the existence of neuronal stretch receptors on the gut that monitor the volume of ingested food is studied in other insects [55,57,58,59], the existence and molecular nature of these receptors in *Drosophila* remains to be established.

#### 1.2.2. Hormonal Communication

Intestinal physiology in *Drosophila* is modulated by extrinsic hormones that are released by endocrine glands such as the ring gland in the brain ((*Drosophila* insulin-like peptide (DILP), Akh, Ast-A, sNPF, neuropeptide F (NPF), corazonin (Crz), leucokinin (Lk)) [60,61,62,63,64,65] or the fat body ((DILP, unpaired 2 (Upd2), CCHa2)) [66,67,68]. The intestine also produces peptide hormones that are also capable of modulating the physiology of the gut. The EE cells-derived hormones are produced by 95% of the total EE cells [69,70,71]. Most of these peptides are also produced by the brain except CCHamide [72].

Neuropeptides are small proteins that act as neuromodulators and regulatory hormones in the CNS and peripheral nervous system (PNS). They are involved in various biological processes such as learning, circadian activity, ecdysis behavior, and feeding [73,74,75]. Out of 30 genes that encode neuropeptides, 7 genes encode insulin-like peptide [76,77]. These peptides bind to G-protein-coupled receptors that are specific to neuropeptides. Each of these neuropeptides is localized in a specific set of neurons and is responsible for the regulation of homeostasis, modulation of neuronal and muscular activity, and coordination of developmental processes.

**Figure 2:**
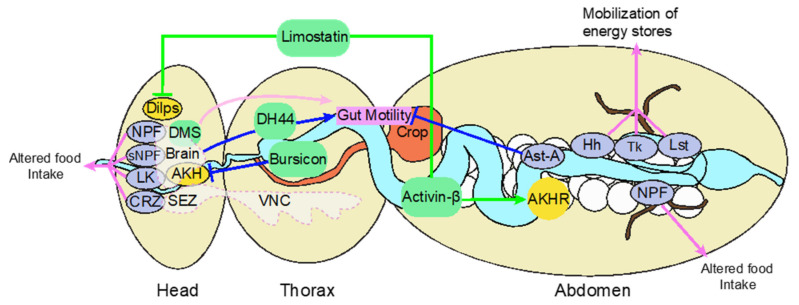
The fly gut is a major organ involved in nutrient-sensing to maintain metabolic homeostasis. The gut secretes multiple hormones and receives information from brain-derived hormones. These signals regulate functions, including gut motility, mobilization, and food intake. Thus, these hormones regulate the nutrients availability. Brain-derived peptides including Crz, DMS, Lk, NPF, sNPF regulate food intake, thereby regulating the quantity of nutrients reaching the GI tract. The gut hormones such as Hh, Tk, Ast-A, Lst, and NPF regulate the amount of food intake, mobilization of energy stores, and gut motility. Bursicon-α derived from EE cells promotes energy storage by inhibiting the production of glucagon-like hormone AKH. Limostatin, secreted by the gut endocrine cell and corpus cardiacum under nutrient-scarce conditions, controls the feeding behavior by suppressing DILP and causes mobilization of the energy via secretion of gut-derived activin-β. VNC is the ventral nerve cord.

The nutritional information sensed by the gut is relayed to produce different responses in the organism depending on the nutrient load. The gut produces signals under nutrient scarcity/starvation and fed conditions that maintain homeostasis in the organism. Gut endocrine neurons secrete neuropeptide, limostatin (Lst) under nutrient-scarce conditions and control the insulin signaling and DILPs secretion (Figure 2) [78]. The EE cells also induce secretion of Tk under starvation conditions that control lipid homeostasis (Figure 2) [79]. Knockdown of Tk is shown to increase intestinal lipogenesis. The nutrients assimilated in the gut are stored and released by the liver as per the requirement to provide energy to other organs. Hh is a nutrient-responsive signal derived from the gut. Hh mobilizes lipid stores and regulate the growth and development of the organism. Hh mutants show increased sensitivity to starvation and fail to mobilize the lipid stores (Figure 2). On the other hand, under fed conditions, the Hh mutants show fast development and pupate early [80]. Therefore, it is considered that Hh is capable of coordinating responses to the availability of nutrients by acting as a lipoprotein-associated endocrine hormone. EE-derived bursicon-α is also known to promote energy storage by restricting the production of glucagon-like hormone AKH [81]. Mobilization of the energy store can also stabilize the level of sugar upon secretion of gut-derived activin-β [82] (Figure 2).

Neuropeptides controlling the feeding behavior of the fruit fly are released from the brain as well as from the gut. The gut-derived peptides are NPF, AST, DH, Tk, and Lst. The peptides derived from CNS are Hugin, NPF, DH, AST, Lk, limostatin, DILP, and AKH (Figure 2). A few of these neuropeptides are discussed briefly in the next section.

##### Tachykinin

Neurons producing neuropeptide Tk are widely distributed throughout the brain and the gut of the fly. They are found in the anterior lobe, pars intercerebralis, central complex, optic lobe, and the dorsolateral protocerebrum of the fly brain [83]. They are also found in the EE cells of the midgut and anterior hindgut in the GI tract of larva and adult [69,70] and also co-localize with other neural peptides [84,85]. The precursor of Tk is also found in the gut of other animals, including cockroaches, stable fly, guinea pig, and rat [86,87,88,89].

In addition to regulating the peristaltic movement in the gut [44], the neuropeptides respond to nutrient availability in the gut by affecting the nutrients stored in the fat body [79]. The signaling also affects the ability of flies to search for food by affecting their olfactory system [90]. In nutrient-scarce conditions in hungry flies, an up-regulation of DTKR (*Drosophila* Tk receptor) is observed. The sensory neurons that contain these DTKR also contain receptors Or42b and Or85a, which may explain the link between Tk and olfaction. Under fed conditions, the expression of DTKR decreases, and insulin receptors activate causing food aversion. Tk also causes lipid mobilization (Figure 2) and affects insulin signaling, which thereafter affects the growth and development of the organism [91,92,93,94]. The production of Tk is activated by the presence of dietary protein and carbohydrates circulating in the lumen of the GI tract [95]. A high-protein diet causes the Tk-producing neurons to secrete growth-blocking peptides from the fat body [96]. Growth-blocking peptides secreted by the fat body act as ligands for EGF receptor (EGFR) in flies. On activation of EGFR by adipose growth-blocking peptides, IPC-connecting neurons (ICN) alleviate the inhibition on the insulin-producing neurons [96]. ICNs with and without modified EGFR signaling produce Tk, which makes it evident that ICN behave as Tk neurons. This phenomenon occurs when the flies are allowed to feed on a high-protein diet. The phenomenon of growth-blocking peptides processing, secretion by adipose tissue, and transportation in the hemolymph is still not clear [96].

##### *Drosophila* Insulin-like Peptide and Adipokinetic Hormone

In fruit flies, DILP’s and AKH share functional similarities to insulin and glucagon found in mammals, respectively [60,97,98,99,100]. The cells secreting these hormones are also functionally analogous to their mammalian counterparts. The insulin-producing cells (IPCs) and AKH-producing cells in the fly brain are analogous to pancreatic beta-cells and alpha-cells found in mammals (Figure 2). There are eight *Drosophila* DILPs. Although many of them are found in the fly brain, DILP 4,5,6 are expressed in the larval midgut [60,69,99], DILP3 in the adult gut, and DILP6 is produced in the fat body (Figure 2) [67,101]. DILP is also present in the gut of other invertebrates [102]. Expression of AKH is observed in the gut of aphids, *Apis mellifera,* and armyworm but not in *Drosophila* [69,103,104,105]. Insulin and glucagon, the counterparts of DILP’s and AKH in humans, are secreted by the pancreas, an accessory digestive organ [106].

The location of IPC- and AKH-producing cells play a major role in the sensation of nutrients, particularly sugars, in the lumen of the gut. The IPCs are located in the median neurosecretory cells in larvae and adults; however, the position of AKH-producing cells differs in different stages of the fly life, conferring to different modes of secretion of hormones [107]. This causes a difference in nutrient-mediated response in larvae and adults. DILPs sense the dietary sugars in the lumen of the gut in adult flies, but in the larvae, the secretion of DILP3 is dependent on AKH [108]. DILPs and AKH both regulate lipid and carbohydrate metabolism. While AKh promotes lipolysis, DILP’s promote lipogenesis [109,110,111].

There are various factors that regulate the IPCs and AKH-producing cells in different stages of the life cycle. The IPCs are regulated by nutrients [112,113,114,115], other neuropeptides [66,78,92,108,116,117,118] and neurotransmitters [119,120,121,122]. The AKH-producing cells are regulated by nutrients [108] and neuropeptides [81,100,118,123]. Nutrients, particularly sugars and fats, stimulate the IPCs via the fat body. The larval fat body produces leptin-like protein, Upd2, which binds to GABAergic neurons and release neurotransmitter GABA, which inhibits the release of DILP from IPCs [112,124]. This indicates indirect regulation by IPCs in larvae. In addition, dietary amino acids also regulate the secretion from IPCs indirectly. It acts via the fat body and release DILP [125]. In adults, the IPC is stimulated by dietary sugars [126,127]. Protein-specific regulation of insulin signaling causes protein-induced feeding inhibition [128]. A high-protein diet causes secretion of DILP that results in reduced feeding in adults [129].

##### Neuropeptide F and Short Neuropeptide F

The two families of NPF and sNPF are present throughout the Arthropoda phylum and are known to coordinate the feeding mechanisms and the metabolism of the insects [45,130]. Both the families are responsible for the intake of food (Figure 2), lipid metabolism, locomotory activity, olfactory behavior, immunity, and circadian rhythm [45,84,131,132,133]. NPF is a functional homolog of the mammalian neuropeptide Y [134]. The larval and adult fly brains express NPF [63,135]. It is also present in the endocrine cells of the midgut (Figure 2) [63]. The subesophageal neurons found in the larval stages of the fly express NPF and respond to sugars [133]. Exposure of larvae to fructose or glucose results in a dose-dependent increase in NPF. These neurons receive gustatory inputs and mediate responses that impact the feeding behaviors of the fly. Silencing NPF ceases the feeding behavior in insects, while overexpression causes extended feeding and delays pupation [136]. NPF binds to its receptor NPFR present in cells of the midgut, ventral nerve cord, and brain (Figure 2) [137]. Activation of NPFR stimulates feeding even on noxious compounds similar to feeding after prolonged starvation [138]. Overexpression of NPFR1 drives fed larvae to feed more under cold conditions [139].

The sNPF neuropeptide is found in the neurons in the hypocerebral ganglion innervating the midgut and innervate fan-shaped body of the central complex in *Drosophila* [84]. It binds to the sNPF receptor, which belongs to G-protein-coupled receptors. There are four sNPF isoforms in flies [140,141]. Due to its role in the regulation of feeding, it is also known as the hunger hormone [28,142,143]. Altered expression of sNPF and sNPFR controls the feeding intake. Down-regulating sNPF suppresses feeding while its overexpression affects the growth of the organism and promotes food intake both in feeding larvae and adults [28]. Similar results are obtained on starvation. Starved flies show an up-regulation of sNPF. The sNPF neuropeptide also regulates osmotic and metabolic stress, locomotion and plays a role in learning, olfaction, and hormone release [84,85,144]. In other insects, such as the cockroach, the midgut shows expression of sNPF [145]; however, no endocrine cells in *Drosophila* have been found to express sNPF [69]. The receptors of sNPF are present on the midgut, hindgut, malpighian tubules, and crop [146].

##### Hugin

Hugin is a neuropeptide co-expressed in the subesophageal ganglion of the adult and larval brain [147,148]. It is homologous to mammalian neuromedin U and is thought to control the initiation of feeding by suppressing the immediate feeding response. Hugin gene is observed to be down-regulated by starvation [149] and also by yeast deprivation in larvae. Over-expressing hugin in flies suppresses the feeding of the larvae. Inhibiting hugin-expressing neurons reduce latency to feeding behavior in adults. It is up-regulated in *pumpless* and *klumpfus* mutant flies that do not show larval feeding behavior [149]. Neuromedin U is expressed commonly in the gut of goldfish and humans [150], but no expression in the fly gut has been documented.

##### Allatostatin

Three families of allatostatin (allatostatin-A (Ast-A), allatostatin-B (Ast-B), and allatostatin-C (Ast-C) [151,152]) are known to inhibit the biosynthesis of juvenile hormone (JH) in corpora allatum in insects [153,154,155]. The Ast-A peptide family does not regulate JH in all insects and instead is involved in suppressing food intake and promoting an aversion to unpalatable food [16,62,118]. Ast-A does not directly regulate the feeding behavior; it is believed that Ast-A acts as an intrinsic factor and then inhibits gut motility [16] (Figure 2). Ast-A is also known to regulate AKH and DILP, thereby affecting the feeding behavior of the flies [118]. Ast-A immunoreactive endocrine cells have been found in the posterior midgut of the fruit fly [69]. Its receptor express in the crop, midgut, hindgut, and the malpighian tubules [69].

##### Leucokinin

Lk is derived from the CNS neurons that terminate at the abdominal wall (Figure 2) [30,65,156,157]. An altered level of Lk expression or its receptor affects the physiology of the gut. Down-regulation of the receptor or the ligand ruptures the abdominal wall and thus leads to fluid retention and abnormal excretion. Leucokinin is also a regulator of the amount of food in the foregut and the termination of the meal (Figure 2). In mutant flies, the flies tend to consume more food in each meal; however, the caloric intake is observed to be constant. This is proposed to be due to the increase in the inter-meal interval, which creates a balance [158]. Leucokinin, unlike other neuropeptides, can function independently. Ablation of other neuropeptides such as hugin and NPF has no effect on the meal size of the fly [158]. The receptors for leucokinin are expressed in the foregut, hindgut, and malpighian tubules [157,158,159].

##### Corazonin

Another neuropeptide affecting the feeding behavior is Crz. Crz is a homolog of mammalian gonadotropin hormone that is commonly expressed in adipocytes and promotes food intake [160]. Crz is produced by brain lateral neurosecretory cells (Figure 2). It is related to AKH, and its receptors are related to the AKH receptor family. Crz receptors are present in the adult salivary glands and in the fat body [161,162,163,164]. While ablation of Crz is known to reduce trehalose level [165], activating Crz-producing neurons results in increased food intake in adults and impacts the triglyceride level [160,165]. Crz is also known to affect lipid and carbohydrate metabolism [116,163,166]. Down-regulation of the Crz receptor results in decreased food intake and also increases the glucose level in hemolymph [163].

##### Other Neuropeptides

Limostatin is secreted by the gut endocrine cells and AKH-producing neurons in the corpus cardiacum under nutrient-scarce conditions [78]. It controls the feeding behavior by suppressing insulin signaling and by mobilizing energy stores (Figure 2). CCHamide is another neuropeptide whose receptor is present on IPCs. CCHamide plays a major role in the feeding, sensory perception, and growth of the organism [66,167,168]. The CCHamide is an orexigenic peptide whose expression is promoted by the nutrients in the gut [66,168]. CCHamide may act by promoting the expression of DILPs and increasing insulin signaling [66,168]. Among CCHamides, CCHa2 is expressed in the brain, fat body, and midgut [66,168]. Dh44 is a homolog of the mammalian corticotropin-releasing hormone (CRH), which gets activated by nutritive sugars. Disturbed activity of Dh44 neurons results in failure to select nutritive sugars [40]. Dh44 relays information to Dh44 receptor R1 neurons in the brain and R2 cells in the gut (Figure 2). Activation of Dh44 neurons suggest its importance for gut motility and excretion in flies [40]. Dh31 is a peptide similar to mammalian calcitonin, which is known to stimulate fluid secretion by malpighian tubules [169]. It is expressed in the posterior midgut [69]. Dromyosuppressin (DMS) is a fly gut peptide (Figure 2) [39] that is expressed in the adult CNS, extending into the rectum, near the anus, and regulates crop motility and contractions [170]. RFAmides are another class of neuropeptides that play a key role in food intake, sensing, and feeding [171]. *Drosophila* contains five genes encoding RFamides, four of these genes are expected to be expressed in the midgut [69]. An important neuropeptide is PDF. PDF synchronizes clock neurons and is expressed in the ventral nerve cord (VNC). PDF neurons innervate the gut of larva and adult fruit flies. This suggests that the neuropeptide PDF and the clock neurons have some unidentified role in feeding and nutrient sensing [33,35,172]. Table 1 summarizes the comparison of human and fly gut discussed so far.

### 1.3. Taste Receptors in Fly Gut

Increasing evidence is proving that the taste receptors and taste signaling molecules are also expressed in the intestinal enteroendocrine cells across vertebrates and invertebrates [198,199]. *Drosophila* taste receptors are spread all over its body, including proboscis, legs, wings, and ovipositor. Gustatory receptors (Grs) help in detecting the appropriate nutrient-rich food and avoiding toxic chemicals. Activation of taste receptor neurons elicits different elements of the feeding program. The structure and functioning of the mammalian intestine and fly gut are similar, and therefore, the expression of Grs in fly gut have been investigated. There are 60 genes in the fly genome that encode for Grs proteins and are believed to be ligand-gated ion channels [200]. Using Gal4/UAS system, 15 Grs (Gr28b.e, Gr33a, Gr36c, Gr39a.a, Gr39a.b, Gr43a, Gr64a, Gr93a, Gr28a, Gr59a, Gr28b.a, Gr28b.b, Gr28b.c, Gr28b.d, Gr58c) are found to be expressed in gut. A total of 12 of these Grs-labeled EE cells in the midgut of flies [199]. It has been shown that EE cells show high activation in the middle midgut with minimal sucrose [95]. Other than sugar, protein cues also lead to the activation of EE cells in the posterior midgut, suggesting a role in the detection of nutrients in the diet, including amino acids specifically. This subset of EE cells also co-express neuropeptides such as DH31 and Tk. These brain-gut peptides are involved in feeding pathways and nutrient-sensing mechanisms [79,95,201]. Gq-coupled calcium-sensing receptor such as CaSR in rats expressed in EE cells is involved in amino acid sensing [202]. Taste cells or EE cells in mammals also produce several peptides that have roles in feeding, satiety, hunger as well as metabolism [203]. They include glucagon, neuropeptide Y, peptide YY, and some others [203]. *Drosophila* has homologous proteins for these and other peptides, which point towards obvious conservation of these peptides and their functions. Apart from certain Grs, the role of other chemosensory receptors, including Pickpocket [204,205,206] genes involved in osmoregulation; Ionotrophic receptors [207,208] known for their role in olfaction, taste [209,210,211,212,213,214,215,216,217,218], and transient receptor potential channels are not known in gut however they are known to modulate feeding preferences via olfactory and gustatory circuits [219,220,221]. Like hunger, thirst is an important internal state, but the role of ppk in the gut, its neural connectivity, the brain, and the type of neurohormones they release are still undetermined.

### 1.4. Nutrient Sensing via Gut in Health and Disease

The number of genes and pathways that play a vital role in metabolic diseases are conserved between flies and humans [222]. Dysregulation of nutrient sensing by the gut has been associated with metabolic syndromes [223,224]. In the past, the major focus on gut studies was to understand the relationship between gut flora and brain disorders because fly intestines have much simpler gut microbiota as opposed to vertebrate intestines [225].

It has been noticed that diet-induced obesity in flies is also associated with many of the pathophysiological consequences found in humans, including hyperglycemia, insulin resistance, cardiac arrhythmia and fibrosis, reduced longevity [226,227], and nephrosis [228]. The gut is important in the absorption of dietary lipid and macronutrients, including sugars, proteins, and fats. It also plays a major role in peripheral body fat storage and metabolizes both glucose and lipids into metabolic intermediates, loading them into hemolymph, which later is used in other tissues and organs. In flies, lipoprotein complexes containing apolipophorins carry sterols and diacylglycerols from the gut to other tissues [229]. Fly lipoproteins also contain Hh, a cholesterol-linked, gut-derived ligand that binds to the transmembrane receptor patched on fat body target cells and promote lipolysis during larval starvation [80,230]. The human anti-obesity drug orlistat, a gastric lipase inhibitor, has been shown to reduce body fat accumulation in adult flies [231]. Supporting a crucial role for lipolysis, midgut lipid accumulation and global fat storage reduce by the insulin signaling pathway inhibitor Foxo in enterocytes via reducing the expression of magro as flies age [232]. Humanized flies show excessive lipid accumulation in the gut and fat body upon expression of human peptide neurotensin in *Drosophila* midgut EE cells, causing obesity, which is triggered by an evolutionarily conserved mechanism acting via the cellular energy sensor 5′ adenosine monophosphate (AMP)-activated protein kinase [233]. Both global vacuolar-type H+ adenosine triphosphatase (ATPase) mutants and flies treated with pharmacological inhibitors of alimentary acidity store extra fat [234], suggesting the role of acidic pH of the gastric lumen in fly obesity. This effect could be mediated via the gut microbiota, which both shapes and depends upon the acidity of the gut [188]. Collectively, these studies emphasize the importance of gut physiology for fat homeostasis in *Drosophila* and highlight possible interactions between the gut epithelium and gut microbiota. Thus, gut microbiota and its metabolism also play an important role in the modulation of fat storage in the fly. Fly gut is enriched in *Lactobacillus* and *Acetobacter* species. Adult axenic flies overstore fats under various dietary conditions [235]. *Lactobacillus* sp. abundance supports co-colonization by *Acetobacter* sp. in the adult gut, which in turn negatively correlates with the fat storage level of the fly [236]. Like other animals, the diet of a fly impacts the composition of the gut microbiota as a high-sugar diet shifts the gut microbiota to uracil-producing species, which promote fat storage and growth in *Drosophila* larvae [237]. The availability of dietary glucose in flies depends on the microbiota because flies with commensal *Acetobacter* tropicalis eat more than axenic flies but store less TAG owing to the consumption of dietary sugar by the bacteria [238]. Taste and olfactory receptors in organs and tissues relevant to metabolic diseases are also summarized in Table 2.

### 1.5. Nutrient Sensing in Cancer and Neurodegenerative Diseases

It has been noticed that intestinal health has a significant impact on neurodegeneration. Microbial dysbiosis [266], dietary changes [267], probiotics [268], and a variety of other disease conditions [269,270] result in the involvement of the gut-brain signaling pathways in the pathophysiology of neurodegenerative diseases. Multiple studies have suggested an association between gut microbiota dysbiosis and the aggregation of amyloid-β (Aβ) peptides in intestinal epithelial cells [271,272] and involvement of CNS [273,274] after high-fat diet feeding. Although many neurodegenerative diseases exhibit accumulation of fibrillary, misfolded proteins similar to the propagation of prionopathies in the CNS [275] but prionopathy also involves GBA and the local immune system, where prions accumulate in dendritic cells in the Peyer’s patches and other lymphoid follicles once entering the intestinal epithelium layer [276]. By interacting with dendritic cells, the misfolded proteins may transport to ENS and ultimately spread to the CNS compartment [276]. The role of gut bacteria in altering peripheral nerve function through the production of neuromodulatory metabolites such as short-chain fatty acid (SCFAs) [277] has been suggested. It has been suggested that oral administration of *Lactobacillus* probiotics shows improvement in the rough-eye phenotype in AD flies [278] and reduces *Wolbachia*’s presence in the gut, which is known to be associated with neurodegenerative disorders. These studies emphasize the relation between gut microbiota, GBA, and the associated neurodegenerative disorders. *Parkin* gene associated with Parkinson’s disease in *Drosophila* [279,280] in ECs is required to maintain the microbial load. The microbial composition in *parkin* mutants is found to be drastically different from wild-type flies. In autism spectrum disorder (ASD), low levels of KDM5 cause intestinal epithelium disruption. The presence of gases produced by the overgrowth of bacteria causes bubble formation in the KDM5 mutant’s midgut and change the composition of gut flora [281]. Though huge attention has been paid to the gut microbiota in neurodegenerative diseases, areas involving gut-brain neural connectivity, abnormal hormonal function, and nutrient sensing have not been explored in this context. Future studies are needed to address the role of neuro hormones in the pathophysiology of neurodegenerative diseases.

The role of the gut in various cancers has been explored, too, using *Drosophila*. The loss of fly adenomatous polyposis coli gene (APC, tumor suppressor gene) causes an increase in ISC proliferation in the gut [282], resembling conditions seen in intestinal adenomas. JNK-Wg signaling regulates the number of gut ISCs. Damaged ECs lead to a surge in JNK signaling and a rise in Wg ligands in (EB) activating JAK-STAT ligands Upd2 and Upd3, which further increase ISCs’ non-autonomous over-proliferation [283]. The absence of APC in ISCs increases the JAK-STAT pathway and affects ISCs proliferation. This helps in establishing the conservation of pathways that regulate ISC proliferation and gut homeostasis [284]. It has been shown that APC and RAS control cell growth in the gut by interacting with one another. In *Drosophila*, orthologous forms of activation of non-receptor tyrosine kinase c-Src lead to ISC proliferation, and inactivation inhibits further ISC proliferation [285]. These results suggest how ISC proliferation is directly involved with cancer formation in both flies to mammals. Oncogenes in the hindgut synergize with the innate immune system to stimulate tumor cell invasion. After bacterial infection, ISCs proliferate but less in the hindgut as opposed to the midgut. RAS oncogene RASv12 induces cell invasion and dissemination of ECs into the abdominal cavity. Upon RASv12expression, hindgut shows a cancerlike phenotype, activating the JNK signaling pathway, which in turn increases ISC proliferation and hence, tumor growth [286]. Studies examining the effect of the altered proliferation of ISCs on nutrient sensing in the gut are still lacking.

## 2. New Research Avenues and Conclusions

*Drosophila* shares a homologous gut system with that of humans, and conservation between mammalian and fly intestinal signaling pathways, pathophysiology, and regeneration that control them makes fly gut an interesting system to study gut nutrient sensing and gut-brain neuronal signaling (Table 1). Fly techniques including vivo CRISPR transcriptional activation (CRISPRa) and interference (CRISPRi) approaches [274,287], single-cell RNA sequencing, direct optogenetics activation of gustatory receptors, molecular neuro-genetics, and behavioral assays provide the complex cellular composition of a real intestine and opportunities to assess various cell types and their physiological roles compared to the mammalian system at a single-cell level. Many parallels have been observed in intestinal mechanisms found in flies to be active in mammals (Table 1) and may therefore become relevant in the context of human pathologies, including diabetes, obesity, neurodegenerative diseases, GI cancers, or aging. Some examples of olfactory and taste receptors found in extra oral and extra nasal tissues relevant to metabolic diseases are highlighted in Table 2. Their diverse functions other than just chemosensory roles show their potential to be therapeutically exploited [288] in health and disease. Parallel findings of communication between gut and brain via different neuropeptides in both humans and flies emphasize the importance of the gut in maintaining health. Neural circuits linking the gut and brain have played a major role in probing the role of the gut in several metabolic disorders, neurological syndromes, aging, and cardiovascular diseases. Study of specific gut regions, various cell types during various developmental stages, stem cell biology, and aging in flies have given clarity to mechanisms not known so far and handle to look at the diseases from a new perspective. Many other functions, including the role of unidentified chemo receptors in the gut, their connectivity with the brain mediating nutrient digestion/transport through neuropeptides, or organs such as the crop and the proventiculus, remain inadequately characterized. Greater understanding is required on the function of the gut microbiota on gut-brain neural circuitry. It is yet to investigate the exact key neurons that mediate nutrient sensing to regulate metabolism and play a key role in human or insect (patho)physiology concerning feeding behavior and appetitive learning. Developing new techniques and behavioral assays can help us explore physiological drives: what is the gut function to maintain the overall health of the animal. They would help to tease apart complexities of gut integrating various metabolites and the role of gut microbiota in nutrient sensing. It would be interesting to find out the key intestinal sensors and nutrient-induced gut signaling to the brain during energy expenditure. How the physical association between gut and brain via neural micro circuits regulate decisions regarding nutrition, hunger and satiety have been poorly characterized. Future work is needed to detail the connections between nutrient sensing and the role of thermosensors and capsacin in accepting incoming hot meals.

Deregulation of nutrient sensing and signaling pathways in the gut not only can affect overall wellbeing and mental health but can therefore lead to faster aging as well. Nutrigenomics studies changes induced by diet on the genome of the animal where health, diet, and genomics are important subjects to consider. *Drosophila* is an ideal model for in vivo modeling for nutrigenomic studies because they have functional orthologs of ~75% of human disease-related genes and organs/tissues that perform the equivalent functions of most mammalian organs, while discrete clusters of cells maintain insect carbohydrate homeostasis in a way similar to pancreatic cells. The mechanistic connections between nutrition sensing via gut and longevity in flies are still underexplored. Moreover, the role of nutrient-sensing mechanisms by the gut in mediating sleep is still lacking.

Animals including *Drosophila* eat and prefer to feed on appetitive substances during particular hours of the day. Since circadian clocks of fly gut cells are subject to signaling cues, including the timing of food intake during the day [289], and play an overarching role in regulating human physiology, it is of prime importance to study clock genes network in the gut during physiological changes that regulate feeding behaviors, sleep, and metabolism. Disruption of circadian rhythms is associated with sleep disorders [290,291] and metabolic syndrome [292]. Understanding genomics of circadian rhythms in gut nutrient sensing will provide opportunities for the discovery of improved treatment strategies and a new understanding of biological underpinnings in human health and disease.

Extensive evidence indicates that regulated nutrient sensing via the gut is the key to keeping metabolic disorders at bay. Dysregulation of nutrient sensing-dependent gut-brain pathways and abnormal gut microbiota in conditions such as obesity has been observed and invites future studies examining the gene and environmental interactions for further development of personalized medicine approaches to treat metabolic diseases. We anticipate that findings from intestinal pathology, gut-brain neural circuitry/signaling, neuropeptides, and conserved biology mechanisms in flies will emphasize the most conserved aspects of human intestinal biology. The current and future fly work on nutrient sensing via gut will contribute greatly to translational research investigating the effect of drugs, microbial and host genetic component analyses, leading to novel findings that are broadly applicable to human health and disease [293].

## Figures and Tables

**Table 1:** Comparison between fly and human intestine.

Category	*Drosophila*	Humans	Reference
Anatomical Comparison	Intestine is made up of epithelial monolayer, which consists of enterocytes (ECs) and enteroendocrine cells.	The intestine contains absorptive ECs and secretory cells, enteroendocrine (EE) cells.	[18,173,174]
	ISCs are present throughout the epithelium. They divide symmetrically and non-symmetrically to give rise to new cells. Wg is required for maintenance of ISCs.	The ISCs show proliferative activity and regenerate new cells. Wnt is responsible for maintaining ISCs.	[17,18,175,176,177]
	Gut is endodermal in origin.	Gut is endodermal in origin.	[178,179]
	Peritrophic matrix is considered to protect the epithelium from abrasive food and microbes.	Mucous layer of the mammalian digestive tract protects the epithelium from abrasive food and microbes.	[180,181]
	Food consumed is stored in crop.	Food is stored in stomach in humans, where partial digestion of food also occurs.	[182]
	Fly fat bodies regulate metabolism, organism homeostasis, growth, and development.	Human adipose tissue regulates metabolism, organism homeostasis, growth, and development.	[183,184,185]
	Malphigian tubules are involved in osmoregulation and excretion and open into the midgut-hindgut junction.	Human kidney is functionally analogous to malphigian tubules.	[10,186]
	*Drosophila* does not have a lamina propria.	Has lamina propria.	[174,175,176,177,178,179,180,181,182,183,184,185,186,187]
Functional Comparison	Digestion in flies occurs under neutral or basic pH conditions.	Mammalian digestion takes place under acidic conditions.	[174,188,189]
	Absorption of nutrients takes place in the anterior midgut.	Absorption of nutrients takes place in the small intestine of humans.	[174]
	Absorption of water and electrolytes occurs in the hindgut.	Absorption of water and electrolytes occurs in large intestine.	[174]
	Adult fly has plenty of microbes in the intestine. Disrupted indigenous microbiota are associated with disease conditions.	Human intestine has plenty of microbes. Disrupted indigenous microbiota are associated with disease conditions.	[190,191,192,193]
Neural Comparison	The GI tract receives innervations from three different sources (stomatogastric nervous system, corpus cardiacum, and neurons of the CNS that extend their axon toward the digestive tract) and relay signals to three distinct regions of the gut (the anterior portion of the gut that comprises of the pharynx, esophagus, crop and the anterior midgut; the midgut-hindgut junction and the posterior hindgut).	Auerbach’s plexus and Meissner’s plexus provide motor and secretomotor innervation to the muscular layer and the mucosal layer in the intestinal lumen, respectively. The vagus nerve acts as the connection between the gut and brain.	[24,25,26,27,28,29,30,31,194,195]
Hormonal Comparison	AKHs regulate lipid and carbohydrate metabolism are produced by AKH-producing cells in the brain. DILPs regulate lipid and carbohydrate metabolism and are produced by IPCs.	Glucagon regulates lipid and carbohydrate metabolism and is produced by pancreatic alpha-cells found in mammals. Insulin regulates lipid and carbohydrate metabolism and is produced by pancreatic beta-cells.	[60,97,98,99,100]
	Upd2 in flies inhibits release of DILP from IPC, thereby affecting the metabolism.	Human leptin alters food intake and energy expenditure.	[112,124,196]
	NPF is known to coordinate the feeding mechanism and the metabolism in the fly.	Neuropeptide Y plays an important role in dietary consumption.	[45,130,134]
	Hugin controls initiation of feeding.	Mammalian neuromedin U plays an important role in gastric acid secretion and feeding behavior.	[147,148,197]
	Nutritive sugars activate Dh44 and have an important role in fly gut motility and excretion.	CRH significantly affects food intake.	[40,196]

**Table 2:** Taste and olfactory receptors in organs and tissues relevant to metabolic diseases.

Olfactory and Taste Receptors with Species	Organ/Tissue	Function	Ligand
OLFR544 (mouse) [239]	Adipocyte (white and brown adipose tissue cells)	Induction of lipolysis and thermogenesis	Azelaic acid
FFAR4 [240,241,242,243,244] (mouse and human)	Adipose tissue (white and brown adipose tissue cells)	Regulation of adipogenesis, promoting browning of white fat, increase in thermogenic activity	Omega-3 PUFAs
TAS2R [245] (mouse)	Adipose tissue (white adipose tissue cells)	Inhibition of adipocyte differentiation	Bitter agonists
TAS2Rs r [246,247,248,249] (mouse and human)	Gastrointestinal tract (EE cells)	Ghrelin, CCK, and GLP1 release	Bitter agonists, berberine, Hoodia gordonii and wild bitter gourd
FFAR1 and FFAR4 [250,251,252,253,254] (mouse and human)	Gastrointestinal tract (EE cells)	Ghrelin, somatostatin, gastrin, CCK, GLP1, and GIP release	FFAR1: medium-chain and long-chain free fatty acids FFAR4: omega-3 PUFAs
TAS1R1–TAS1R3 [255,256,257,258] (mouse and human)	Gastrointestinal tract (EE cells)	Ghrelin, CCK, and GLP1 release	Amino acids and oligopeptides
TAS1R2–TAS1R3 [255,259,260] (mouse and human)	Gastrointestinal tract (EE cells)	GLP1 release	Glucose (antagonist lactisole)
TAS2R [261] (human)	Gastrointestinal tract (parietal cells)	Stimulation of gastric acid secretion	Bitter agonists
TAS1R2–TAS1R3 [262]	Pancreas (β-cells)	Potentiation of glucose-induced insulin release by fructose and artificial sweeteners	Sweet agonists
FFAR1 [263,264] (rodent and human)	Pancreas (β-cells)	Contradictory findings: potentiation of glucose-induced insulin secretion but also impaired glucose homeostasis	Medium-chain and long-chain fatty acids
FFAR4 [265] (mouse and human)	Pancreas (δ-cells)	Inhibition of somatostatin to regulate insulin secretion	Omega-3 PUFAs

## Abbreviations

ENS	Enteric nervous system
CNS	Central nervous system
GI	Gastrointestinal
ISCs	Intestinal stem cells
ECs	Enterocytes
EE	Enteroendocrine
EBs	Enteroblasts
GBA	Gut-brain axis
VNC	Ventral nerve cord
PDF	Pigment dispersing factor
ITP	Ion transport peptide
Akh	Adipokinetic hormone
AST	Allatostatin
Dh44	Diuretic hormone 44
sNPF	Short neuropeptide F
Tk	Tachykinin
HGN1	Hindgut neuron1
IN1	Interneurons neurons
Ilp7	Insulin-like peptide 7
Hh	Hedgehog
DILP2	*Drosophila* insulin-like peptide
PNS	Peripheral nervous system
Lst	Limostatin
NPF	Neuropeptide F
Lk	Leucokinin
DTKR	Drosophila Tk receptor
IPCs	Insulin-producing cell
Upd2	Unpaired 2
EGFR	EGF receptor
ICN	IPC-connecting neurons
Ast-A	Allatostatin-A
Ast-B	Allatostatin-B
Ast-C	Allatostatin-C
Crz	Corazonin
DTKR	*Drosophila* Tk receptor
JH	Juvenile hormone
CRH	Corticotropin-releasing hormone
DMS	Dromyosuppressin
AMP	Adenosine monophosphate
ATPase	Adenosine triphosphatase
ASD	Autism spectrum disorder
Aβ	Amyloid β
SCFAs	Short-chain fatty acid
APC	Adenomatous polyposis coli gene
CRISPRa	CRISPR transcriptional activation
CRISPRi	CRISPR interference
TAS1R	Taste receptor family 1
OLFR544	Olfactory receptor 544
FFAR	Free fatty acid receptor
GLP1	Glucagon-like peptide 1
PUFA	Polyunsaturated fatty acid
CCK	Cholecystokinin
GIP	Gastric inhibitory polypeptide

## References

[1] Thompson D.G., Malagelada J.R. (1981). Guts and their motions (gastrointestinal motility in health and disease). J. Clin. Gastroenterol..

[2] Bentsen M.A., Mirzadeh Z., Schwartz M.W. (2019). Revisiting How the Brain Senses Glucose—And Why. Cell Metab..

[3] Rutter G.A., Pullen T.J., Hodson D.J., Martinez-Sanchez A. (2015). Pancreatic beta-cell identity, glucose sensing and the control of insulin secretion. Biochem. J..

[4] Oosterveer M.H., Schoonjans K. (2014). Hepatic glucose sensing and integrative pathways in the liver. Cell. Mol. Life Sci..

[5] Gartner L.P. (1985). The fine structural morphology of the midgut of adult Drosophila: A morphometric analysis. Tissue. Cell.

[6] King D.G. (1988). Cellular organization and peritrophic membrane formation in the cardia (proventriculus) of *Drosophila melanogaster*. J. Morphol..

[7] Murakami R., Shiotsuki Y. (2001). Ultrastructure of the hindgut of *Drosophila larvae*, with special reference to the domains identified by specific gene expression patterns. J. Morphol..

[8] Marianes A., Spradling A.C. (2013). Physiological and stem cell compartmentalization within the *Drosophila* midgut. eLife.

[9] Murakami R., Shigenaga A., Matsumoto A., Yamaoka I., Tanimura T. (1994). Novel tissue units of regional differentiation in the gut epithelium of *Drosopbila*, as revealed by P-element-mediated detection of enhancer. Roux’s Arch. Dev. Biol..

[10] Dow J.A., Maddrell S.H., Gortz A., Skaer N.J., Brogan S., Kaiser K. (1994). The malpighian tubules of *Drosophila melanogaster*: A novel phenotype for studies of fluid secretion and its control. J. Exp. Biol..

[11] Mathur D., Bost A., Driver I., Ohlstein B. (2010). A transient niche regulates the specification of *Drosophila* intestinal stem cells. Science.

[12] Takashima S., Hartenstein V. (2012). Genetic control of intestinal stem cell specification and development: A comparative view. Stem. Cell Rev. Rep..

[13] Takashima S., Adams K.L., Ortiz P.A., Ying C.T., Moridzadeh R., Younossi-Hartenstein A., Hartenstein V. (2011). Development of the *Drosophila* entero-endocrine lineage and its specification by the Notch signaling pathway. Dev. Biol..

[14] Lemaitre B., Miguel-Aliaga I. (2013). The digestive tract of *Drosophila melanogaster*. Annu. Rev. Genet..

[15] Raybould H.E. (1998). Does Your Gut Taste? Sensory Transduction in the Gastrointestinal Tract. News Physiol. Sci..

[16] Chen J., Reiher W., Hermann-Luibl C., Sellami A., Cognigni P., Kondo S., Helfrich-Forster C., Veenstra J.A., Wegener C. (2016). Allatostatin A Signalling in *Drosophila* Regulates Feeding and Sleep and Is Modulated by PDF. PLoS Genet..

[17] Micchelli C.A., Perrimon N. (2006). Evidence that stem cells reside in the adult *Drosophila* midgut epithelium. Nature.

[18] Ohlstein B., Spradling A. (2006). The adult *Drosophila* posterior midgut is maintained by pluripotent stem cells. Nature.

[19] Apidianakis Y., Pitsouli C., Perrimon N., Rahme L. (2009). Synergy between bacterial infection and genetic predisposition in intestinal dysplasia. Proc. Natl. Acad. Sci. USA.

[20] Jiang H., Patel P.H., Kohlmaier A., Grenley M.O., McEwen D.G., Edgar B.A. (2009). Cytokine/Jak/Stat signaling mediates regeneration and homeostasis in the *Drosophila* midgut. Cell.

[21] Biteau B., Karpac J., Supoyo S., Degennaro M., Lehmann R., Jasper H. (2010). Lifespan extension by preserving proliferative homeostasis in *Drosophila*. PLoS Genet..

[22] Buchon N., Broderick N.A., Poidevin M., Pradervand S., Lemaitre B. (2009). *Drosophila* intestinal response to bacterial infection: Activation of host defense and stem cell proliferation. Cell Host Microbe.

[23] Da Lage J.L., Maczkowiak F., Cariou M.L. (2000). Molecular characterization and evolution of the amylase multigene family of *Drosophila ananassae*. J. Mol. Evol..

[24] Hartenstein V., Tepass U., Gruszynski-Defeo E. (1994). Embryonic development of the stomatogastric nervous system in *Drosophila*. J. Comp. Neurol..

[25] Gonzalez-Gaitan M., Jackle H. (1995). Invagination centers within the *Drosophila* stomatogastric nervous system anlage are positioned by Notch-mediated signaling which is spatially controlled through wingless. Development.

[26] Pankratz M.J., Hoch M. (1995). Control of epithelial morphogenesis by cell signaling and integrin molecules in the *Drosophila* foregut. Development.

[27] Spiess R., Schoofs A., Heinzel H.G. (2008). Anatomy of the stomatogastric nervous system associated with the foregut in *Drosophila melanogaster* and *Calliphora vicina* third instar larvae. J. Morphol..

[28] Lee K.S., You K.H., Choo J.K., Han Y.M., Yu K. (2004). *Drosophila* short neuropeptide F regulates food intake and body size. J. Biol. Chem..

[29] Miguel-Aliaga I., Thor S. (2004). Segment-specific prevention of pioneer neuron apoptosis by cell-autonomous, postmitotic Hox gene activity. Development.

[30] Cognigni P., Bailey A.P., Miguel-Aliaga I. (2011). Enteric neurons and systemic signals couple nutritional and reproductive status with intestinal homeostasis. Cell Metab..

[31] Schoofs A., Huckesfeld S., Surendran S., Pankratz M.J. (2014). Serotonergic pathways in the *Drosophila* larval enteric nervous system. J. Insect Physiol..

[32] Miguel-Aliaga I., Thor S., Gould A.P. (2008). Postmitotic specification of *Drosophila* insulinergic neurons from pioneer neurons. PLoS Biol..

[33] Talsma A.D., Christov C.P., Terriente-Felix A., Linneweber G.A., Perea D., Wayland M., Shafer O.T., Miguel-Aliaga I. (2012). Remote control of renal physiology by the intestinal neuropeptide pigment-dispersing factor in *Drosophila*. Proc. Natl. Acad. Sci. USA.

[34] Anderson M.S., Halpern M.E., Keshishian H. (1988). Identification of the neuropeptide transmitter proctolin in *Drosophila* larvae: Characterization of muscle fiber-specific neuromuscular endings. J. Neurosci..

[35] Nassel D.R., Shiga S., Mohrherr C.J., Rao K.R. (1993). Pigment-dispersing hormone-like peptide in the nervous system of the flies *Phormia* and *Drosophila*: Immunocytochemistry and partial characterization. J. Comp. Neurol..

[36] Dircksen H., Tesfai L.K., Albus C., Nassel D.R. (2008). Ion transport peptide splice forms in central and peripheral neurons throughout postembryogenesis of *Drosophila melanogaster*. J. Comp. Neurol..

[37] Dircksen H. (2009). Insect ion transport peptides are derived from alternatively spliced genes and differentially expressed in the central and peripheral nervous system. J. Exp. Biol..

[38] Budnik V., Wu C.F., White K. (1989). Altered branching of serotonin-containing neurons in Drosophila mutants unable to synthesize serotonin and dopamine. J. Neurosci..

[39] McCormick J., Nichols R. (1993). Spatial and temporal expression identify dromyosuppressin as a brain-gut peptide in *Drosophila melanogaster*. J. Comp. Neurol..

[40] Dus M., Lai J.S., Gunapala K.M., Min S., Tayler T.D., Hergarden A.C., Geraud E., Joseph C.M., Suh G.S. (2015). Nutrient Sensor in the Brain Directs the Action of the Brain-Gut Axis in *Drosophila*. Neuron.

[41] Miyamoto T., Slone J., Song X., Amrein H. (2012). A fructose receptor functions as a nutrient sensor in the *Drosophila* brain. Cell.

[42] Mishra D., Miyamoto T., Rezenom Y.H., Broussard A., Yavuz A., Slone J., Russell D.H., Amrein H. (2013). The molecular basis of sugar sensing in *Drosophila* larvae. Curr. Biol..

[43] Miyamoto T., Amrein H. (2014). Diverse roles for the *Drosophila* fructose sensor Gr43a. Fly.

[44] Siviter R.J., Coast G.M., Winther A.M., Nachman R.J., Taylor C.A., Shirras A.D., Coates D., Isaac R.E., Nassel D.R. (2000). Expression and functional characterization of a *Drosophila* neuropeptide precursor with homology to mammalian preprotachykinin A. J. Biol. Chem..

[45] Lee G., Park J.H. (2004). Hemolymph sugar homeostasis and starvation-induced hyperactivity affected by genetic manipulations of the adipokinetic hormone-encoding gene in *Drosophila melanogaster*. Genetics.

[46] Nichols R., Bendena W.G., Tobe S.S. (2002). Myotropic peptides in *Drosophila melanogaster* and the genes that encode them. J. Neurogenet..

[47] Kenmoku H., Ishikawa H., Ote M., Kuraishi T., Kurata S. (2016). A subset of neurons controls the permeability of the peritrophic matrix and midgut structure in *Drosophila* adults. J. Exp. Biol..

[48] Linneweber G.A., Jacobson J., Busch K.E., Hudry B., Christov C.P., Dormann D., Yuan M., Otani T., Knust E., de Bono M. (2014). Neuronal control of metabolism through nutrient-dependent modulation of tracheal branching. Cell.

[49] Wayland M.T., Defaye A., Rocha J., Jayaram S.A., Royet J., Miguel-Aliaga I., Leulier F., Cognigni P. (2014). Spotting the differences: Probing host/microbiota interactions with a dedicated software tool for the analysis of faecal outputs in *Drosophila*. J. Insect Physiol..

[50] Yapici N., Cohn R., Schusterreiter C., Ruta V., Vosshall L.B. (2016). A Taste Circuit that Regulates Ingestion by Integrating Food and Hunger Signals. Cell.

[51] Olds W.H., Xu T. (2014). Regulation of food intake by mechanosensory ion channels in enteric neurons. eLife.

[52] Zhang W., Yan Z., Li B., Jan L.Y., Jan Y.N. (2014). Identification of motor neurons and a mechanosensitive sensory neuron in the defecation circuitry of *Drosophila* larvae. eLife.

[53] Han H., Pan C., Liu C., Lv X., Yang X., Xiong Y., Lu Y., Wu W., Han J., Zhou Z. (2015). Gut-neuron interaction via Hh signaling regulates intestinal progenitor cell differentiation in *Drosophila*. Cell Discov..

[54] Min S., Oh Y., Verma P., Whitehead S.C., Yapici N., Van Vactor D., Suh G.S., Liberles S. (2021). Control of feeding by Piezo-mediated gut mechanosensation in *Drosophila*. eLife.

[55] Dethier V.G., Gelperin A. (1967). Hyperphagia in the Blowfly. J. Exp. Biol..

[56] Pool A.H., Kvello P., Mann K., Cheung S.K., Gordon M.D., Wang L., Scott K. (2014). Four GABAergic interneurons impose feeding restraint in *Drosophila*. Neuron.

[57] Stoffolano J.G., Haselton A.T. (2013). The adult Dipteran crop: A unique and overlooked organ. Annu. Rev. Entomol..

[58] Chapman R.F. (1998). The Insects: Structure and Function.

[59] Belzer W.R. (1978). Recurrent nerve inhibition of protein feeding in the blowfly *Phormia regina*. Physiol. Entomol..

[60] Ikeya T., Galic M., Belawat P., Nairz K., Hafen E. (2002). Nutrient-dependent expression of insulin-like peptides from neuroendocrine cells in the CNS contributes to growth regulation in *Drosophila*. Curr. Biol..

[61] Oh Y., Lai J.S., Mills H.J., Erdjument-Bromage H., Giammarinaro B., Saadipour K., Wang J.G., Abu F., Neubert T.A., Suh G.S.B. (2019). A glucose-sensing neuron pair regulates insulin and glucagon in *Drosophila*. Nature.

[62] Hergarden A.C., Tayler T.D., Anderson D.J. (2012). Allatostatin-A neurons inhibit feeding behavior in adult *Drosophila*. Proc. Natl. Acad. Sci. USA.

[63] Brown M.R., Crim J.W., Arata R.C., Cai H.N., Chun C., Shen P. (1999). Identification of a Drosophila brain-gut peptide related to the neuropeptide Y family. Peptides.

[64] Imura E., Shimada-Niwa Y., Nishimura T., Huckesfeld S., Schlegel P., Ohhara Y., Kondo S., Tanimoto H., Cardona A., Pankratz M.J. (2020). The Corazonin-PTTH Neuronal Axis Controls Systemic Body Growth by Regulating Basal Ecdysteroid Biosynthesis in *Drosophila melanogaster*. Curr. Biol..

[65] de Haro M., Al-Ramahi I., Benito-Sipos J., Lopez-Arias B., Dorado B., Veenstra J.A., Herrero P. (2010). Detailed analysis of leucokinin-expressing neurons and their candidate functions in the *Drosophila* nervous system. Cell Tissue Res..

[66] Sano H., Nakamura A., Texada M.J., Truman J.W., Ishimoto H., Kamikouchi A., Nibu Y., Kume K., Ida T., Kojima M. (2015). The Nutrient-Responsive Hormone CCHamide-2 Controls Growth by Regulating Insulin-Like Peptides in the Brain of *Drosophila melanogaster*. PLoS Genet..

[67] Okamoto N., Yamanaka N., Yagi Y., Nishida Y., Kataoka H., O’Connor M.B., Mizoguchi A. (2009). A fat body-derived IGF-like peptide regulates postfeeding growth in *Drosophila*. Dev. Cell.

[68] Ingaramo M.C., Sanchez J.A., Perrimon N., Dekanty A. (2020). Fat Body p53 Regulates Systemic Insulin Signaling and Autophagy under Nutrient Stress via *Drosophila* Upd2 Repression. Cell Rep..

[69] Veenstra J.A., Agricola H.J., Sellami A. (2008). Regulatory peptides in fruit fly midgut. Cell Tissue Res..

[70] Veenstra J.A. (2009). Peptidergic paracrine and endocrine cells in the midgut of the fruit fly maggot. Cell Tissue Res..

[71] Reiher W., Shirras C., Kahnt J., Baumeister S., Isaac R.E., Wegener C. (2011). Peptidomics and peptide hormone processing in the *Drosophila* midgut. J. Proteome Res..

[72] Li S., Torre-Muruzabal T., Sogaard K.C., Ren G.R., Hauser F., Engelsen S.M., Podenphanth M.D., Desjardins A., Grimmelikhuijzen C.J. (2013). Expression patterns of the *Drosophila* neuropeptide CCHamide-2 and its receptor may suggest hormonal signaling from the gut to the brain. PLoS ONE.

[73] Ewer J., Frisch B., Hamblen-Coyle M.J., Rosbash M., Hall J.C. (1992). Expression of the period clock gene within different cell types in the brain of *Drosophila* adults and mosaic analysis of these cells’ influence on circadian behavioral rhythms. J. Neurosci..

[74] Kingan T.G., Zitnan D., Jaffe H., Beckage N.E. (1997). Identification of neuropeptides in the midgut of parasitized insects: FLRFamides as candidate paracrines. Mol. Cell. Endocrinol..

[75] Zitnan D., Sehnal F., Bryant P.J. (1993). Neurons producing specific neuropeptides in the central nervous system of normal and pupariation-delayed *Drosophila*. Dev. Biol..

[76] Adams M.D., Celniker S.E., Holt R.A., Evans C.A., Gocayne J.D., Amanatides P.G., Scherer S.E., Li P.W., Hoskins R.A., Galle R.F. (2000). The genome sequence of *Drosophila melanogaster*. Science.

[77] Hewes R.S., Taghert P.H. (2001). Neuropeptides and neuropeptide receptors in the *Drosophila melanogaster* genome. Genome Res..

[78] Alfa R.W., Park S., Skelly K.R., Poffenberger G., Jain N., Gu X., Kockel L., Wang J., Liu Y., Powers A.C. (2015). Suppression of insulin production and secretion by a decretin hormone. Cell Metab..

[79] Song W., Veenstra J.A., Perrimon N. (2014). Control of lipid metabolism by tachykinin in *Drosophila*. Cell Rep..

[80] Rodenfels J., Lavrynenko O., Ayciriex S., Sampaio J.L., Carvalho M., Shevchenko A., Eaton S. (2014). Production of systemically circulating Hedgehog by the intestine couples nutrition to growth and development. Genes Dev..

[81] Scopelliti A., Bauer C., Yu Y., Zhang T., Kruspig B., Murphy D.J., Vidal M., Maddocks O.D.K., Cordero J.B. (2019). A Neuronal Relay Mediates a Nutrient Responsive Gut/Fat Body Axis Regulating Energy Homeostasis in Adult *Drosophila*. Cell Metab..

[82] Song W., Cheng D., Hong S., Sappe B., Hu Y., Wei N., Zhu C., O’Connor M.B., Pissios P., Perrimon N. (2017). Midgut-Derived Activin Regulates Glucagon-Like Action in the Fat Body and Glycemic Control. Cell Metab..

[83] Winther A.M., Siviter R.J., Isaac R.E., Predel R., Nassel D.R. (2003). Neuronal expression of tachykinin-related peptides and gene transcript during postembryonic development of *Drosophila*. J. Comp. Neurol..

[84] Kahsai L., Martin J.R., Winther A.M. (2010). Neuropeptides in the *Drosophila* central complex in modulation of locomotor behavior. J. Exp. Biol..

[85] Carlsson M.A., Diesner M., Schachtner J., Nassel D.R. (2010). Multiple neuropeptides in the *Drosophila* antennal lobe suggest complex modulatory circuits. J. Comp. Neurol..

[86] Brookes S.J., Song Z.M., Steele P.A., Costa M. (1992). Identification of motor neurons to the longitudinal muscle of the guinea pig ileum. Gastroenterology.

[87] Khan I., Collins S.M. (1994). Fourth isoform of preprotachykinin messenger RNA encoding for substance P in the rat intestine. Biochem. Biophys. Res. Commun..

[88] Andriès J.C., Tramu G. (1985). Ultrastructural and immunohistochemical study of endocrine cells in the midgut of the cockroach *Blaberus craniifer* (Insecta, Dictyoptera). Cell Tissue Res..

[89] Guerrero F.D. (1997). Transcriptional expression of a putative tachykinin-like peptide receptor gene from stable fly. Peptides.

[90] Ko K.I., Root C.M., Lindsay S.A., Zaninovich O.A., Shepherd A.K., Wasserman S.A., Kim S.M., Wang J.W. (2015). Starvation promotes concerted modulation of appetitive olfactory behavior via parallel neuromodulatory circuits. eLife.

[91] Kamareddine L., Robins W.P., Berkey C.D., Mekalanos J.J., Watnick P.I. (2018). The *Drosophila* Immune Deficiency Pathway Modulates Enteroendocrine Function and Host Metabolism. Cell Metab..

[92] Birse R.T., Soderberg J.A., Luo J., Winther A.M., Nassel D.R. (2011). Regulation of insulin-producing cells in the adult *Drosophila* brain via the tachykinin peptide receptor DTKR. J. Exp. Biol..

[93] Poels J., Birse R.T., Nachman R.J., Fichna J., Janecka A., Vanden Broeck J., Nassel D.R. (2009). Characterization and distribution of NKD, a receptor for *Drosophila* tachykinin-related peptide 6. Peptides.

[94] Amcheslavsky A., Song W., Li Q., Nie Y., Bragatto I., Ferrandon D., Perrimon N., Ip Y.T. (2014). Enteroendocrine cells support intestinal stem-cell-mediated homeostasis in *Drosophila*. Cell Rep..

[95] Park J.H., Chen J., Jang S., Ahn T.J., Kang K., Choi M.S., Kwon J.Y. (2016). A subset of enteroendocrine cells is activated by amino acids in the *Drosophila* midgut. FEBS Lett..

[96] Meschi E., Leopold P., Delanoue R. (2019). An EGF-Responsive Neural Circuit Couples Insulin Secretion with Nutrition in *Drosophila*. Dev. Cell.

[97] Wang S., Tulina N., Carlin D.L., Rulifson E.J. (2007). The origin of islet-like cells in *Drosophila* identifies parallels to the vertebrate endocrine axis. Proc. Natl. Acad. Sci. USA.

[98] Kim S.K., Rulifson E.J. (2004). Conserved mechanisms of glucose sensing and regulation by *Drosophila* corpora cardiaca cells. Nature.

[99] Brogiolo W., Stocker H., Ikeya T., Rintelen F., Fernandez R., Hafen E. (2001). An evolutionarily conserved function of the *Drosophila* insulin receptor and insulin-like peptides in growth control. Curr. Biol..

[100] Rulifson E.J., Kim S.K., Nusse R. (2002). Ablation of insulin-producing neurons in flies: Growth and diabetic phenotypes. Science.

[101] Slaidina M., Delanoue R., Gronke S., Partridge L., Leopold P. (2009). A *Drosophila* insulin-like peptide promotes growth during nonfeeding states. Dev. Cell.

[102] Lecroisey C., Le Petillon Y., Escriva H., Lammert E., Laudet V. (2015). Identification, evolution and expression of an insulin-like peptide in the cephalochordate *Branchiostoma lanceolatum*. PLoS ONE.

[103] Abdel-Latief M., Hoffmann K.H. (2007). The adipokinetic hormones in the fall armyworm, *Spodoptera frugiperda*: cDNA cloning, quantitative real time RT-PCR analysis, and gene specific localization. Insect Biochem. Mol. Biol..

[104] Christie A.E. (2020). Assessment of midgut enteroendocrine peptide complement in the honey bee, *Apis mellifera*. Insect Biochem. Mol. Biol..

[105] Jedlickova V., Jedlicka P., Lee H.J. (2015). Characterization and expression analysis of adipokinetic hormone and its receptor in eusocial aphid *Pseudoregma bambucicola*. Gen. Comp. Endocrinol..

[106] Gosmain Y., Cheyssac C., Heddad Masson M., Dibner C., Philippe J. (2011). Glucagon gene expression in the endocrine pancreas: The role of the transcription factor Pax6 in alpha-cell differentiation, glucagon biosynthesis and secretion. Diabetes Obes. Metab..

[107] Dai J.D., Gilbert L.I. (1991). Metamorphosis of the corpus allatum and degeneration of the prothoracic glands during the larval-pupal-adult transformation of *Drosophila melanogaster*: A cytophysiological analysis of the ring gland. Dev. Biol..

[108] Kim J., Neufeld T.P. (2015). Dietary sugar promotes systemic TOR activation in *Drosophila* through AKH-dependent selective secretion of Dilp3. Nat. Commun..

[109] Toprak U. (2020). The Role of Peptide Hormones in Insect Lipid Metabolism. Front. Physiol..

[110] Gronke S., Muller G., Hirsch J., Fellert S., Andreou A., Haase T., Jackle H., Kuhnlein R.P. (2007). Dual lipolytic control of body fat storage and mobilization in *Drosophila*. PLoS Biol..

[111] Choi S., Lim D.S., Chung J. (2015). Feeding and Fasting Signals Converge on the LKB1-SIK3 Pathway to Regulate Lipid Metabolism in *Drosophila*. PLoS Genet..

[112] Rajan A., Perrimon N. (2012). *Drosophila* cytokine unpaired 2 regulates physiological homeostasis by remotely controlling insulin secretion. Cell.

[113] Colombani J., Raisin S., Pantalacci S., Radimerski T., Montagne J., Leopold P. (2003). A nutrient sensor mechanism controls *Drosophila* growth. Cell.

[114] Delanoue R., Meschi E., Agrawal N., Mauri A., Tsatskis Y., McNeill H., Leopold P. (2016). *Drosophila* insulin release is triggered by adipose Stunted ligand to brain Methuselah receptor. Science.

[115] Koyama T., Mirth C.K. (2016). Growth-Blocking Peptides as Nutrition-Sensitive Signals for Insulin Secretion and Body Size Regulation. PLoS Biol..

[116] Kapan N., Lushchak O.V., Luo J., Nassel D.R. (2012). Identified peptidergic neurons in the *Drosophila* brain regulate insulin-producing cells, stress responses and metabolism by coexpressed short neuropeptide F and corazonin. Cell. Mol. Life Sci..

[117] Lee K.S., Kwon O.Y., Lee J.H., Kwon K., Min K.J., Jung S.A., Kim A.K., You K.H., Tatar M., Yu K. (2008). *Drosophila* short neuropeptide F signalling regulates growth by ERK-mediated insulin signalling. Nat. Cell Biol..

[118] Hentze J.L., Carlsson M.A., Kondo S., Nassel D.R., Rewitz K.F. (2015). The Neuropeptide Allatostatin A Regulates Metabolism and Feeding Decisions in *Drosophila*. Sci. Rep..

[119] Andreatta G., Kyriacou C.P., Flatt T., Costa R. (2018). Aminergic Signaling Controls Ovarian Dormancy in *Drosophila*. Sci. Rep..

[120] Kaplan D.D., Zimmermann G., Suyama K., Meyer T., Scott M.P. (2008). A nucleostemin family GTPase, NS3, acts in serotonergic neurons to regulate insulin signaling and control body size. Genes Dev..

[121] Luo J., Becnel J., Nichols C.D., Nassel D.R. (2012). Insulin-producing cells in the brain of adult *Drosophila* are regulated by the serotonin 5-HT1A receptor. Cell. Mol. Life Sci..

[122] Luo J., Lushchak O.V., Goergen P., Williams M.J., Nassel D.R. (2014). *Drosophila* insulin-producing cells are differentially modulated by serotonin and octopamine receptors and affect social behavior. PLoS ONE.

[123] Post S., Liao S., Yamamoto R., Veenstra J.A., Nassel D.R., Tatar M. (2019). *Drosophila* insulin-like peptide dilp1 increases lifespan and glucagon-like Akh expression epistatic to dilp2. Aging Cell.

[124] Enell L.E., Kapan N., Soderberg J.A., Kahsai L., Nassel D.R. (2010). Insulin signaling, lifespan and stress resistance are modulated by metabotropic GABA receptors on insulin producing cells in the brain of *Drosophila*. PLoS ONE.

[125] Geminard C., Rulifson E.J., Leopold P. (2009). Remote control of insulin secretion by fat cells in *Drosophila*. Cell Metab..

[126] Park S., Alfa R.W., Topper S.M., Kim G.E., Kockel L., Kim S.K. (2014). A genetic strategy to measure circulating *Drosophila* insulin reveals genes regulating insulin production and secretion. PLoS Genet..

[127] Kreneisz O., Chen X., Fridell Y.W., Mulkey D.K. (2010). Glucose increases activity and Ca^2+^ in insulin-producing cells of adult *Drosophila*. Neuroreport.

[128] Sun J., Liu C., Bai X., Li X., Li J., Zhang Z., Zhang Y., Guo J., Li Y. (2017). *Drosophila* FIT is a protein-specific satiety hormone essential for feeding control. Nat. Commun..

[129] Semaniuk U.V., Gospodaryov D.V., Feden’ko K.M., Yurkevych I.S., Vaiserman A.M., Storey K.B., Simpson S.J., Lushchak O. (2018). Insulin-Like Peptides Regulate Feeding Preference and Metabolism in *Drosophila*. Front. Physiol..

[130] Mirabeau O., Joly J.S. (2013). Molecular evolution of peptidergic signaling systems in bilaterians. Proc. Natl. Acad. Sci. USA.

[131] Johard H.A., Yoishii T., Dircksen H., Cusumano P., Rouyer F., Helfrich-Forster C., Nassel D.R. (2009). Peptidergic clock neurons in *Drosophila*: Ion transport peptide and short neuropeptide F in subsets of dorsal and ventral lateral neurons. J. Comp. Neurol..

[132] Shen R., Wang B., Giribaldi M.G., Ayres J., Thomas J.B., Montminy M. (2016). Neuronal energy-sensing pathway promotes energy balance by modulating disease tolerance. Proc. Natl. Acad. Sci. USA.

[133] Shen P., Cai H.N. (2001). *Drosophila* neuropeptide F mediates integration of chemosensory stimulation and conditioning of the nervous system by food. J. Neurobiol..

[134] Fadda M., Hasakiogullari I., Temmerman L., Beets I., Zels S., Schoofs L. (2019). Regulation of Feeding and Metabolism by Neuropeptide F and Short Neuropeptide F in Invertebrates. Front. Endocrinol..

[135] Krashes M.J., DasGupta S., Vreede A., White B., Armstrong J.D., Waddell S. (2009). A neural circuit mechanism integrating motivational state with memory expression in *Drosophila*. Cell.

[136] Wu Q., Wen T., Lee G., Park J.H., Cai H.N., Shen P. (2003). Developmental control of foraging and social behavior by the *Drosophila* neuropeptide Y-like system. Neuron.

[137] Garczynski S.F., Brown M.R., Shen P., Murray T.F., Crim J.W. (2002). Characterization of a functional neuropeptide F receptor from *Drosophila melanogaster*. Peptides.

[138] Wu Q., Zhang Y., Xu J., Shen P. (2005). Regulation of hunger-driven behaviors by neural ribosomal S6 kinase in *Drosophila*. Proc. Natl. Acad. Sci. USA.

[139] Lingo P.R., Zhao Z., Shen P. (2007). Co-regulation of cold-resistant food acquisition by insulin- and neuropeptide Y-like systems in *Drosophila melanogaster*. Neuroscience.

[140] Vanden Broeck J. (2001). Neuropeptides and their precursors in the fruitfly, *Drosophila melanogaster*. Peptides.

[141] Baggerman G., Cerstiaens A., De Loof A., Schoofs L. (2002). Peptidomics of the larval *Drosophila melanogaster* central nervous system. J. Biol. Chem..

[142] Root C.M., Ko K.I., Jafari A., Wang J.W. (2011). Presynaptic facilitation by neuropeptide signaling mediates odor-driven food search. Cell.

[143] Sudhakar S.R., Pathak H., Rehman N., Fernandes J., Vishnu S., Varghese J. (2020). Insulin signalling elicits hunger-induced feeding in *Drosophila*. Dev. Biol..

[144] Nassel D.R., Elekes K. (1992). Aminergic neurons in the brain of blowflies and *Drosophila*: Dopamine- and tyrosine hydroxylase-immunoreactive neurons and their relationship with putative histaminergic neurons. Cell Tissue Res..

[145] Veenstra J.A., Lau G.W., Agricola H.J., Petzel D.H. (1995). Immunohistological localization of regulatory peptides in the midgut of the female mosquito *Aedes aegypti*. Histochem. Cell Biol..

[146] Mertens I., Meeusen T., Huybrechts R., De Loof A., Schoofs L. (2002). Characterization of the short neuropeptide F receptor from *Drosophila melanogaster*. Biochem. Biophys. Res. Commun..

[147] Bader R., Colomb J., Pankratz B., Schrock A., Stocker R.F., Pankratz M.J. (2007). Genetic dissection of neural circuit anatomy underlying feeding behavior in Drosophila: Distinct classes of hugin-expressing neurons. J. Comp. Neurol..

[148] Bader R., Wegener C., Pankratz M.J. (2007). Comparative neuroanatomy and genomics of hugin and pheromone biosynthesis activating neuropeptide (PBAN). Fly.

[149] Melcher C., Pankratz M.J. (2005). Candidate gustatory interneurons modulating feeding behavior in the *Drosophila* brain. PLoS Biol..

[150] Maruyama K., Kaiya H., Miyazato M., Murakami N., Nakahara K., Matsuda K. (2019). Purification and identification of native forms of goldfish neuromedin U from brain and gut. Biochem. Biophys. Res. Commun..

[151] Lenz C., Williamson M., Grimmelikhuijzen C.J. (2000). Molecular cloning and genomic organization of an allatostatin preprohormone from *Drosophila melanogaster*. Biochem. Biophys. Res. Commun..

[152] Williamson M., Lenz C., Winther A.M., Nassel D.R., Grimmelikhuijzen C.J. (2001). Molecular cloning, genomic organization, and expression of a B-type (cricket-type) allatostatin preprohormone from *Drosophila melanogaster*. Biochem. Biophys. Res. Commun..

[153] Kramer S.J., Toschi A., Miller C.A., Kataoka H., Quistad G.B., Li J.P., Carney R.L., Schooley D.A. (1991). Identification of an allatostatin from the tobacco hornworm *Manduca sexta*. Proc. Natl. Acad. Sci. USA.

[154] Lorenz M.W., Kellner R., Hoffmann K.H. (1995). A family of neuropeptides that inhibit juvenile hormone biosynthesis in the cricket, *Gryllus bimaculatus*. J. Biol. Chem..

[155] Belles X., Graham L.A., Bendena W.G., Ding Q.I., Edwards J.P., Weaver R.J., Tobe S.S. (1999). The molecular evolution of the allatostatin precursor in cockroaches. Peptides.

[156] O’Donnell M.J., Rheault M.R., Davies S.A., Rosay P., Harvey B.J., Maddrell S.H., Kaiser K., Dow J.A. (1998). Hormonally controlled chloride movement across *Drosophila* tubules is via ion channels in stellate cells. Am. J. Physiol..

[157] Radford J.C., Davies S.A., Dow J.A. (2002). Systematic G-protein-coupled receptor analysis in *Drosophila melanogaster* identifies a leucokinin receptor with novel roles. J. Biol. Chem..

[158] Al-Anzi B., Armand E., Nagamei P., Olszewski M., Sapin V., Waters C., Zinn K., Wyman R.J., Benzer S. (2010). The leucokinin pathway and its neurons regulate meal size in *Drosophila*. Curr. Biol..

[159] Zandawala M., Yurgel M.E., Texada M.J., Liao S., Rewitz K.F., Keene A.C., Nassel D.R. (2018). Modulation of *Drosophila* post-feeding physiology and behavior by the neuropeptide leucokinin. PLoS Genet..

[160] Zhao Y., Bretz C.A., Hawksworth S.A., Hirsh J., Johnson E.C. (2010). Corazonin neurons function in sexually dimorphic circuitry that shape behavioral responses to stress in *Drosophila*. PLoS ONE.

[161] Veenstra J.A. (1994). Isolation and structure of the *Drosophila* corazonin gene. Biochem. Biophys. Res. Commun..

[162] Sha K., Choi S.H., Im J., Lee G.G., Loeffler F., Park J.H. (2014). Regulation of ethanol-related behavior and ethanol metabolism by the Corazonin neurons and Corazonin receptor in *Drosophila melanogaster*. PLoS ONE.

[163] Kubrak O.I., Lushchak O.V., Zandawala M., Nassel D.R. (2016). Systemic corazonin signalling modulates stress responses and metabolism in *Drosophila*. Open Biol..

[164] Park Y., Kim Y.J., Adams M.E. (2002). Identification of G protein-coupled receptors for *Drosophila* PRXamide peptides, CCAP, corazonin, and AKH supports a theory of ligand-receptor coevolution. Proc. Natl. Acad. Sci. USA.

[165] Lee G., Kim K.M., Kikuno K., Wang Z., Choi Y.J., Park J.H. (2008). Developmental regulation and functions of the expression of the neuropeptide corazonin in *Drosophila melanogaster*. Cell Tissue Res..

[166] Gáliková M., Klepsatel P., Xu Y., Kühnlein R.P. (2017). The obesity-related Adipokinetic hormone controls feeding and expression of neuropeptide regulators of *Drosophila* metabolism. Eur. J. Lipid Sci. Technol..

[167] Farhan A., Gulati J., Grobetae-Wilde E., Vogel H., Hansson B.S., Knaden M. (2013). The CCHamide 1 receptor modulates sensory perception and olfactory behavior in starved *Drosophila*. Sci. Rep..

[168] Ren G.R., Hauser F., Rewitz K.F., Kondo S., Engelbrecht A.F., Didriksen A.K., Schjott S.R., Sembach F.E., Li S., Sogaard K.C. (2015). CCHamide-2 Is an Orexigenic Brain-Gut Peptide in *Drosophila*. PLoS ONE.

[169] Coast G.M., Webster S.G., Schegg K.M., Tobe S.S., Schooley D.A. (2001). The *Drosophila melanogaster* homologue of an insect calcitonin-like diuretic peptide stimulates V-ATPase activity in fruit fly Malpighian tubules. J. Exp. Biol..

[170] Richer S., Stoffolano J.G., Yin C.M., Nichols R. (2000). Innervation of dromyosuppressin (DMS) immunoreactive processes and effect of DMS and benzethonium chloride on the *Phormia regina* (Meigen) crop. J. Comp. Neurol..

[171] Bechtold D.A., Luckman S.M. (2007). The role of RFamide peptides in feeding. J. Endocrinol..

[172] Helfrich-Forster C. (1997). Development of pigment-dispersing hormone-immunoreactive neurons in the nervous system of *Drosophila melanogaster*. J. Comp. Neurol..

[173] Crosnier C., Stamataki D., Lewis J. (2006). Organizing cell renewal in the intestine: Stem cells, signals and combinatorial control. Nat. Rev. Genet..

[174] Capo F., Wilson A., Di Cara F. (2019). The Intestine of *Drosophila melanogaster*: An Emerging Versatile Model System to Study Intestinal Epithelial Homeostasis and Host-Microbial Interactions in Humans. Microorganisms.

[175] Barker N., van Es J.H., Kuipers J., Kujala P., van den Born M., Cozijnsen M., Haegebarth A., Korving J., Begthel H., Peters P.J. (2007). Identification of stem cells in small intestine and colon by marker gene Lgr5. Nature.

[176] Lin G., Xu N., Xi R. (2008). Paracrine Wingless signalling controls self-renewal of *Drosophila* intestinal stem cells. Nature.

[177] Scoville D.H., Sato T., He X.C., Li L. (2008). Current view: Intestinal stem cells and signaling. Gastroenterology.

[178] Kedinger M., Simon-Assmann P., Haffen K. (1987). Growth and differentiation of intestinal endodermal cells in a coculture system. Gut.

[179] Tepass U., Hartenstein V. (1994). Epithelium formation in the *Drosophila* midgut depends on the interaction of endoderm and mesoderm. Development.

[180] Gooday G.W. (1999). Aggressive and defensive roles for chitinases. EXS.

[181] Vodovar N., Vinals M., Liehl P., Basset A., Degrouard J., Spellman P., Boccard F., Lemaitre B. (2005). *Drosophila* host defense after oral infection by an entomopathogenic *Pseudomonas* species. Proc. Natl. Acad. Sci. USA.

[182] Edgecomb R.S., Harth C.E., Schneiderman A.M. (1994). Regulation of feeding behavior in adult *Drosophila melanogaster* varies with feeding regime and nutritional state. J. Exp. Biol..

[183] Zheng H., Yang X., Xi Y. (2016). Fat body remodeling and homeostasis control in *Drosophila*. Life Sci..

[184] Yamada T., Habara O., Kubo H., Nishimura T. (2018). Fat body glycogen serves as a metabolic safeguard for the maintenance of sugar levels in *Drosophila*. Development.

[185] Schoettl T., Fischer I.P., Ussar S. (2018). Heterogeneity of adipose tissue in development and metabolic function. J. Exp. Biol..

[186] Demerec M. (1950). Biology of *Drosophila*.

[187] Mowat A.M., Agace W.W. (2014). Regional specialization within the intestinal immune system. Nat. Rev. Immunol..

[188] Overend G., Luo Y., Henderson L., Douglas A.E., Davies S.A., Dow J.A. (2016). Molecular mechanism and functional significance of acid generation in the *Drosophila* midgut. Sci. Rep..

[189] Shanbhag S., Tripathi S. (2009). Epithelial ultrastructure and cellular mechanisms of acid and base transport in the *Drosophila* midgut. J. Exp. Biol..

[190] Cox C.R., Gilmore M.S. (2007). Native microbial colonization of *Drosophila melanogaster* and its use as a model of *Enterococcus faecalis* pathogenesis. Infect. Immun..

[191] Ryu J.H., Kim S.H., Lee H.Y., Bai J.Y., Nam Y.D., Bae J.W., Lee D.G., Shin S.C., Ha E.M., Lee W.J. (2008). Innate immune homeostasis by the homeobox gene caudal and commensal-gut mutualism in *Drosophila*. Science.

[192] Frank D.N., Pace N.R. (2008). Gastrointestinal microbiology enters the metagenomics era. Curr. Opin. Gastroenterol..

[193] Qin J., Li R., Raes J., Arumugam M., Burgdorf K.S., Manichanh C., Nielsen T., Pons N., Levenez F., Yamada T. (2010). A human gut microbial gene catalogue established by metagenomic sequencing. Nature.

[194] Baumgarten H.G., Holstein A.F., Owman C. (1970). Auerbach’s plexus of mammals and man: Electron microscopic identification of three different types of neuronal processes in myenteric ganglia of the large intestine from rhesus monkeys, guinea-pigs and man. Z. Zellforsch. Mikrosk. Anat..

[195] Bennett A. (1974). Relation between gut motility and innervation in man. Digestion.

[196] Valassi E., Scacchi M., Cavagnini F. (2008). Neuroendocrine control of food intake. Nutr. Metab. Cardiovasc. Dis..

[197] Brighton P.J., Szekeres P.G., Willars G.B. (2004). Neuromedin U and its receptors: Structure, function, and physiological roles. Pharmacol. Rev..

[198] Dyer J., Salmon K.S., Zibrik L., Shirazi-Beechey S.P. (2005). Expression of sweet taste receptors of the T1R family in the intestinal tract and enteroendocrine cells. Biochem. Soc. Trans..

[199] Park J.H., Kwon J.Y. (2011). Heterogeneous expression of *Drosophila* gustatory receptors in enteroendocrine cells. PLoS ONE.

[200] Sato K., Tanaka K., Touhara K. (2011). Sugar-regulated cation channel formed by an insect gustatory receptor. Proc. Natl. Acad. Sci. USA.

[201] Palamiuc L., Noble T., Witham E., Ratanpal H., Vaughan M., Srinivasan S. (2017). A tachykinin-like neuroendocrine signalling axis couples central serotonin action and nutrient sensing with peripheral lipid metabolism. Nat. Commun..

[202] Mace O.J., Schindler M., Patel S. (2012). The regulation of K- and L-cell activity by GLUT2 and the calcium-sensing receptor CasR in rat small intestine. J. Physiol..

[203] Depoortere I. (2014). Taste receptors of the gut: Emerging roles in health and disease. Gut.

[204] Liu L., Leonard A.S., Motto D.G., Feller M.A., Price M.P., Johnson W.A., Welsh M.J. (2003). Contribution of *Drosophila* DEG/ENaC genes to salt taste. Neuron.

[205] Cameron P., Hiroi M., Ngai J., Scott K. (2010). The molecular basis for water taste in *Drosophila*. Nature.

[206] Chen Z., Wang Q., Wang Z. (2010). The amiloride-sensitive epithelial Na^+^ channel PPK28 is essential for *Drosophila* gustatory water reception. J. Neurosci..

[207] Benton R., Vannice K.S., Gomez-Diaz C., Vosshall L.B. (2009). Variant ionotropic glutamate receptors as chemosensory receptors in *Drosophila*. Cell.

[208] Croset V., Rytz R., Cummins S.F., Budd A., Brawand D., Kaessmann H., Gibson T.J., Benton R. (2010). Ancient protostome origin of chemosensory ionotropic glutamate receptors and the evolution of insect taste and olfaction. PLoS Genet..

[209] Croset V., Schleyer M., Arguello J.R., Gerber B., Benton R. (2016). A molecular and neuronal basis for amino acid sensing in the *Drosophila* larva. Sci. Rep..

[210] Hussain A., Zhang M., Ucpunar H.K., Svensson T., Quillery E., Gompel N., Ignell R., Grunwald Kadow I.C. (2016). Ionotropic Chemosensory Receptors Mediate the Taste and Smell of Polyamines. PLoS Biol..

[211] Ganguly A., Pang L., Duong V.K., Lee A., Schoniger H., Varady E., Dahanukar A. (2017). A Molecular and Cellular Context-Dependent Role for Ir76b in Detection of Amino Acid Taste. Cell Rep..

[212] Abuin L., Bargeton B., Ulbrich M.H., Isacoff E.Y., Kellenberger S., Benton R. (2011). Functional architecture of olfactory ionotropic glutamate receptors. Neuron.

[213] Ai M., Min S., Grosjean Y., Leblanc C., Bell R., Benton R., Suh G.S. (2010). Acid sensing by the *Drosophila* olfactory system. Nature.

[214] Ai M., Blais S., Park J.Y., Min S., Neubert T.A., Suh G.S. (2013). Ionotropic glutamate receptors IR64a and IR8a form a functional odorant receptor complex in vivo in *Drosophila*. J. Neurosci..

[215] Silbering A.F., Rytz R., Grosjean Y., Abuin L., Ramdya P., Jefferis G.S., Benton R. (2011). Complementary function and integrated wiring of the evolutionarily distinct *Drosophila* olfactory subsystems. J. Neurosci..

[216] Koh T.W., He Z., Gorur-Shandilya S., Menuz K., Larter N.K., Stewart S., Carlson J.R. (2014). The *Drosophila* IR20a clade of ionotropic receptors are candidate taste and pheromone receptors. Neuron.

[217] Joseph R.M., Sun J.S., Tam E., Carlson J.R. (2017). A receptor and neuron that activate a circuit limiting sucrose consumption. eLife.

[218] Grosjean Y., Rytz R., Farine J.P., Abuin L., Cortot J., Jefferis G.S., Benton R. (2011). An olfactory receptor for food-derived odours promotes male courtship in *Drosophila*. Nature.

[219] Zhang Y.V., Ni J., Montell C. (2013). The molecular basis for attractive salt-taste coding in *Drosophila*. Science.

[220] Kwon Y., Kim S.H., Ronderos D.S., Lee Y., Akitake B., Woodward O.M., Guggino W.B., Smith D.P., Montell C. (2010). *Drosophila* TRPA1 channel is required to avoid the naturally occurring insect repellent citronellal. Curr. Biol..

[221] Badsha F., Kain P., Prabhakar S., Sundaram S., Padinjat R., Rodrigues V., Hasan G. (2012). Mutants in *Drosophila* TRPC channels reduce olfactory sensitivity to carbon dioxide. PLoS ONE.

[222] Reiter L.T., Potocki L., Chien S., Gribskov M., Bier E. (2001). A systematic analysis of human disease-associated gene sequences in *Drosophila melanogaster*. Genome Res..

[223] de Lartigue G., de La Serre C.B., Raybould H.E. (2011). Vagal afferent neurons in high fat diet-induced obesity; intestinal microflora, gut inflammation and cholecystokinin. Physiol. Behav..

[224] Grasset E., Puel A., Charpentier J., Collet X., Christensen J.E., Terce F., Burcelin R. (2017). A Specific Gut Microbiota Dysbiosis of Type 2 Diabetic Mice Induces GLP-1 Resistance through an Enteric NO-Dependent and Gut-Brain Axis Mechanism. Cell Metab..

[225] Broderick N.A., Lemaitre B. (2012). Gut-associated microbes of *Drosophila melanogaster*. Gut Microbes.

[226] Birse R.T., Choi J., Reardon K., Rodriguez J., Graham S., Diop S., Ocorr K., Bodmer R., Oldham S. (2010). High-fat-diet-induced obesity and heart dysfunction are regulated by the TOR pathway in *Drosophila*. Cell Metab..

[227] Na J., Musselman L.P., Pendse J., Baranski T.J., Bodmer R., Ocorr K., Cagan R. (2013). A *Drosophila* model of high sugar diet-induced cardiomyopathy. PLoS Genet..

[228] Na J., Sweetwyne M.T., Park A.S., Susztak K., Cagan R.L. (2015). Diet-Induced Podocyte Dysfunction in *Drosophila* and Mammals. Cell Rep..

[229] Palm W., Sampaio J.L., Brankatschk M., Carvalho M., Mahmoud A., Shevchenko A., Eaton S. (2012). Lipoproteins in *Drosophila melanogaster*—Assembly, function, and influence on tissue lipid composition. PLoS Genet..

[230] Palm W., Swierczynska M.M., Kumari V., Ehrhart-Bornstein M., Bornstein S.R., Eaton S. (2013). Secretion and signaling activities of lipoprotein-associated hedgehog and non-sterol-modified hedgehog in flies and mammals. PLoS Biol..

[231] Sieber M.H., Thummel C.S. (2009). The DHR96 nuclear receptor controls triacylglycerol homeostasis in *Drosophila*. Cell Metab..

[232] Karpac J., Biteau B., Jasper H. (2013). Misregulation of an adaptive metabolic response contributes to the age-related disruption of lipid homeostasis in *Drosophila*. Cell Rep..

[233] Li J., Song J., Zaytseva Y.Y., Liu Y., Rychahou P., Jiang K., Starr M.E., Kim J.T., Harris J.W., Yiannikouris F.B. (2016). An obligatory role for neurotensin in high-fat-diet-induced obesity. Nature.

[234] Lin W.S., Huang C.W., Song Y.S., Yen J.H., Kuo P.C., Yeh S.R., Lin H.Y., Fu T.F., Wu M.S., Wang H.D. (2015). Reduced Gut Acidity Induces an Obese-Like Phenotype in *Drosophila melanogaster* and in Mice. PLoS ONE.

[235] Wong A.C., Dobson A.J., Douglas A.E. (2014). Gut microbiota dictates the metabolic response of *Drosophila* to diet. J. Exp. Biol..

[236] Newell P.D., Douglas A.E. (2014). Interspecies interactions determine the impact of the gut microbiota on nutrient allocation in *Drosophila melanogaster*. Appl. Environ. Microbiol..

[237] Whon T.W., Shin N.R., Jung M.J., Hyun D.W., Kim H.S., Kim P.S., Bae J.W. (2017). Conditionally Pathogenic Gut Microbes Promote Larval Growth by Increasing Redox-Dependent Fat Storage in High-Sugar Diet-Fed *Drosophila*. Antioxid. Redox Signal..

[238] Huang J.H., Douglas A.E. (2015). Consumption of dietary sugar by gut bacteria determines *Drosophila* lipid content. Biol. Lett..

[239] Wu C., Hwang S.H., Jia Y., Choi J., Kim Y.J., Choi D., Pathiraja D., Choi I.G., Koo S.H., Lee S.J. (2017). Olfactory receptor 544 reduces adiposity by steering fuel preference toward fats. J. Clin. Investig..

[240] Quesada-Lopez T., Cereijo R., Turatsinze J.V., Planavila A., Cairo M., Gavalda-Navarro A., Peyrou M., Moure R., Iglesias R., Giralt M. (2016). The lipid sensor GPR120 promotes brown fat activation and FGF21 release from adipocytes. Nat. Commun..

[241] Oh D.Y., Talukdar S., Bae E.J., Imamura T., Morinaga H., Fan W., Li P., Lu W.J., Watkins S.M., Olefsky J.M. (2010). GPR120 is an omega-3 fatty acid receptor mediating potent anti-inflammatory and insulin-sensitizing effects. Cell.

[242] Oh D.Y., Walenta E., Akiyama T.E., Lagakos W.S., Lackey D., Pessentheiner A.R., Sasik R., Hah N., Chi T.J., Cox J.M. (2014). A Gpr120-selective agonist improves insulin resistance and chronic inflammation in obese mice. Nat. Med..

[243] Ichimura A., Hirasawa A., Poulain-Godefroy O., Bonnefond A., Hara T., Yengo L., Kimura I., Leloire A., Liu N., Iida K. (2012). Dysfunction of lipid sensor GPR120 leads to obesity in both mouse and human. Nature.

[244] Abbott K.A., Burrows T.L., Thota R.N., Acharya S., Garg M.L. (2016). Do omega-3 PUFAs affect insulin resistance in a sex-specific manner? A systematic review and meta-analysis of randomized controlled trials. Am. J. Clin. Nutr..

[245] Avau B., Bauters D., Steensels S., Vancleef L., Laermans J., Lesuisse J., Buyse J., Lijnen H.R., Tack J., Depoortere I. (2015). The Gustatory Signaling Pathway and Bitter Taste Receptors Affect the Development of Obesity and Adipocyte Metabolism in Mice. PLoS ONE.

[246] Janssen S., Laermans J., Verhulst P.J., Thijs T., Tack J., Depoortere I. (2011). Bitter taste receptors and alpha-gustducin regulate the secretion of ghrelin with functional effects on food intake and gastric emptying. Proc. Natl. Acad. Sci. USA.

[247] Yu Y., Hao G., Zhang Q., Hua W., Wang M., Zhou W., Zong S., Huang M., Wen X. (2015). Berberine induces GLP-1 secretion through activation of bitter taste receptor pathways. Biochem. Pharmacol..

[248] Le Neve B., Foltz M., Daniel H., Gouka R. (2010). The steroid glycoside H.g.-12 from Hoodia gordonii activates the human bitter receptor TAS2R14 and induces CCK release from HuTu-80 cells. Am. J. Physiol. Gastrointest. Liver Physiol..

[249] Huang T.N., Lu K.N., Pai Y.P., Chin H., Huang C.J. (2013). Role of GLP-1 in the Hypoglycemic Effects of Wild Bitter Gourd. Evid.-Based Complement. Altern. Med..

[250] Janssen S., Laermans J., Iwakura H., Tack J., Depoortere I. (2012). Sensing of fatty acids for octanoylation of ghrelin involves a gustatory G-protein. PLoS ONE.

[251] Engelstoft M.S., Park W.M., Sakata I., Kristensen L.V., Husted A.S., Osborne-Lawrence S., Piper P.K., Walker A.K., Pedersen M.H., Nohr M.K. (2013). Seven transmembrane G protein-coupled receptor repertoire of gastric ghrelin cells. Mol. Metab..

[252] Liou A.P., Lu X., Sei Y., Zhao X., Pechhold S., Carrero R.J., Raybould H.E., Wank S. (2011). The G-protein-coupled receptor GPR40 directly mediates long-chain fatty acid-induced secretion of cholecystokinin. Gastroenterology.

[253] Edfalk S., Steneberg P., Edlund H. (2008). Gpr40 is expressed in enteroendocrine cells and mediates free fatty acid stimulation of incretin secretion. Diabetes.

[254] Tanaka T., Katsuma S., Adachi T., Koshimizu T.A., Hirasawa A., Tsujimoto G. (2008). Free fatty acids induce cholecystokinin secretion through GPR120. Naunyn Schmiedeberg’s Arch. Pharmacol..

[255] Jang H.J., Kokrashvili Z., Theodorakis M.J., Carlson O.D., Kim B.J., Zhou J., Kim H.H., Xu X., Chan S.L., Juhaszova M. (2007). Gut-expressed gustducin and taste receptors regulate secretion of glucagon-like peptide-1. Proc. Natl. Acad. Sci. USA.

[256] Vancleef L., Thijs T., Baert F., Ceulemans L.J., Canovai E., Wang Q., Steensels S., Segers A., Farre R., Pirenne J. (2018). Obesity Impairs Oligopeptide/Amino Acid-Induced Ghrelin Release and Smooth Muscle Contractions in the Human Proximal Stomach. Mol. Nutr. Food Res..

[257] Vancleef L., Van Den Broeck T., Thijs T., Steensels S., Briand L., Tack J., Depoortere I. (2015). Chemosensory signalling pathways involved in sensing of amino acids by the ghrelin cell. Sci. Rep..

[258] Daly K., Al-Rammahi M., Moran A., Marcello M., Ninomiya Y., Shirazi-Beechey S.P. (2013). Sensing of amino acids by the gut-expressed taste receptor T1R1-T1R3 stimulates CCK secretion. Am. J. Physiol. Gastrointest. Liver Physiol..

[259] Gerspach A.C., Steinert R.E., Schonenberger L., Graber-Maier A., Beglinger C. (2011). The role of the gut sweet taste receptor in regulating GLP-1, PYY, and CCK release in humans. Am. J. Physiol. Endocrinol. Metab..

[260] Steinert R.E., Gerspach A.C., Gutmann H., Asarian L., Drewe J., Beglinger C. (2011). The functional involvement of gut-expressed sweet taste receptors in glucose-stimulated secretion of glucagon-like peptide-1 (GLP-1) and peptide YY (PYY). Clin. Nutr..

[261] Liszt K.I., Ley J.P., Lieder B., Behrens M., Stoger V., Reiner A., Hochkogler C.M., Kock E., Marchiori A., Hans J. (2017). Caffeine induces gastric acid secretion via bitter taste signaling in gastric parietal cells. Proc. Natl. Acad. Sci. USA.

[262] Kyriazis G.A., Soundarapandian M.M., Tyrberg B. (2012). Sweet taste receptor signaling in beta cells mediates fructose-induced potentiation of glucose-stimulated insulin secretion. Proc. Natl. Acad. Sci. USA.

[263] Itoh Y., Kawamata Y., Harada M., Kobayashi M., Fujii R., Fukusumi S., Ogi K., Hosoya M., Tanaka Y., Uejima H. (2003). Free fatty acids regulate insulin secretion from pancreatic beta cells through GPR40. Nature.

[264] Steneberg P., Rubins N., Bartoov-Shifman R., Walker M.D., Edlund H. (2005). The FFA receptor GPR40 links hyperinsulinemia, hepatic steatosis, and impaired glucose homeostasis in mouse. Cell Metab..

[265] Stone V.M., Dhayal S., Brocklehurst K.J., Lenaghan C., Sorhede Winzell M., Hammar M., Xu X., Smith D.M., Morgan N.G. (2014). GPR120 (FFAR4) is preferentially expressed in pancreatic delta cells and regulates somatostatin secretion from murine islets of Langerhans. Diabetologia.

[266] Vangay P., Ward T., Gerber J.S., Knights D. (2015). Antibiotics, pediatric dysbiosis, and disease. Cell Host Microbe.

[267] Muegge B.D., Kuczynski J., Knights D., Clemente J.C., Gonzalez A., Fontana L., Henrissat B., Knight R., Gordon J.I. (2011). Diet drives convergence in gut microbiome functions across mammalian phylogeny and within humans. Science.

[268] Delzenne N.M., Neyrinck A.M., Cani P.D. (2011). Modulation of the gut microbiota by nutrients with prebiotic properties: Consequences for host health in the context of obesity and metabolic syndrome. Microb. Cell Fact..

[269] Tilg H., Moschen A.R. (2014). Microbiota and diabetes: An evolving relationship. Gut.

[270] Rosenfeld C.S. (2015). Microbiome Disturbances and Autism Spectrum Disorders. Drug Metab. Dispos..

[271] Galloway S., Takechi R., Pallebage-Gamarallage M.M., Dhaliwal S.S., Mamo J.C. (2009). Amyloid-beta colocalizes with apolipoprotein B in absorptive cells of the small intestine. Lipids Health Dis..

[272] Galloway S., Jian L., Johnsen R., Chew S., Mamo J.C. (2007). Beta-amyloid or its precursor protein is found in epithelial cells of the small intestine and is stimulated by high-fat feeding. J. Nutr. Biochem..

[273] Nam K.N., Mounier A., Wolfe C.M., Fitz N.F., Carter A.Y., Castranio E.L., Kamboh H.I., Reeves V.L., Wang J., Han X. (2017). Effect of high fat diet on phenotype, brain transcriptome and lipidome in Alzheimer’s model mice. Sci. Rep..

[274] Lin B., Hasegawa Y., Takane K., Koibuchi N., Cao C., Kim-Mitsuyama S. (2016). High-Fat-Diet Intake Enhances Cerebral Amyloid Angiopathy and Cognitive Impairment in a Mouse Model of Alzheimer’s Disease, Independently of Metabolic Disorders. J. Am. Heart Assoc..

[275] Goedert M. (2015). NEURODEGENERATION. Alzheimer’s and Parkinson’s diseases: The prion concept in relation to assembled Abeta, tau, and alpha-synuclein. Science.

[276] Ano Y., Sakudo A., Nakayama H., Onodera T. (2009). Uptake and dynamics of infectious prion protein in the intestine. Protein Pept. Lett..

[277] Kimura I., Inoue D., Maeda T., Hara T., Ichimura A., Miyauchi S., Kobayashi M., Hirasawa A., Tsujimoto G. (2011). Short-chain fatty acids and ketones directly regulate sympathetic nervous system via G protein-coupled receptor 41 (GPR41). Proc. Natl. Acad. Sci. USA.

[278] Tan F.H.P., Liu G., Lau S.A., Jaafar M.H., Park Y.H., Azzam G., Li Y., Liong M.T. (2020). Lactobacillus probiotics improved the gut microbiota profile of a *Drosophila melanogaster* Alzheimer’s disease model and alleviated neurodegeneration in the eye. Benef. Microbes.

[279] Feltzin V., Wan K.H., Celniker S.E., Bonini N.M. (2019). Role and impact of the gut microbiota in a *Drosophila* model for parkinsonism. bioRxiv.

[280] Scheperjans F., Aho V., Pereira P.A., Koskinen K., Paulin L., Pekkonen E., Haapaniemi E., Kaakkola S., Eerola-Rautio J., Pohja M. (2015). Gut microbiota are related to Parkinson’s disease and clinical phenotype. Mov. Disord..

[281] Chen K., Luan X., Liu Q., Wang J., Chang X., Snijders A.M., Mao J.H., Secombe J., Dan Z., Chen J.H. (2019). *Drosophila* Histone Demethylase KDM5 Regulates Social Behavior through Immune Control and Gut Microbiota Maintenance. Cell Host Microbe.

[282] Yu X., Waltzer L., Bienz M. (1999). A new *Drosophila* APC homologue associated with adhesive zones of epithelial cells. Nat. Cell Biol..

[283] Tian A., Benchabane H., Ahmed Y. (2018). Wingless/Wnt Signaling in Intestinal Development, Homeostasis, Regeneration and Tumorigenesis: A *Drosophila* Perspective. J. Dev. Biol..

[284] Cordero J.B., Stefanatos R.K., Myant K., Vidal M., Sansom O.J. (2012). Non-autonomous crosstalk between the Jak/Stat and Egfr pathways mediates Apc1-driven intestinal stem cell hyperplasia in the *Drosophila* adult midgut. Development.

[285] Cordero J.B., Ridgway R.A., Valeri N., Nixon C., Frame M.C., Muller W.J., Vidal M., Sansom O.J. (2014). c-Src drives intestinal regeneration and transformation. EMBO J..

[286] Christofi T., Apidianakis Y. (2013). Ras-oncogenic *Drosophila* hindgut but not midgut cells use an inflammation-like program to disseminate to distant sites. Gut Microbes.

[287] Ghosh S., Tibbit C., Liu J.L. (2016). Effective knockdown of *Drosophila* long non-coding RNAs by CRISPR interference. Nucleic Acids Res..

[288] Lee S.J., Depoortere I., Hatt H. (2019). Therapeutic potential of ectopic olfactory and taste receptors. Nat. Rev. Drug Discov..

[289] Parasram K., Bernardon N., Hammoud M., Chang H., He L., Perrimon N., Karpowicz P. (2018). Intestinal Stem Cells Exhibit Conditional Circadian Clock Function. Stem Cell. Rep..

[290] Dutta D., Buchon N., Xiang J., Edgar B.A. (2015). Regional Cell Specific RNA Expression Profiling of FACS Isolated *Drosophila* Intestinal Cell Populations. Curr. Protoc. Stem Cell Biol..

[291] Reynolds S.E. (1997). The biology of the insect midgut. Edited by M.J. Lehane and P.F. Billingsley. London: Chapman & Hall, 1996. xvi + 486 pp. Hard cover £50.00. ISBN 412 61670X. Bull. Entomol. Res..

[292] Mattila J., Havula E., Suominen E., Teesalu M., Surakka I., Hynynen R., Kilpinen H., Vaananen J., Hovatta I., Kakela R. (2015). Mondo-Mlx Mediates Organismal Sugar Sensing through the Gli-Similar Transcription Factor Sugarbabe. Cell Rep..

[293] Sadaqat Z., Kaushik S., Kain P. (2021). Gut Feeding the Brain: *Drosophila* Gut an Animal Model for Medicine to Understand Mechanisms Mediating Food Preferences. Animal Models in Medicine.

